# Foam-Templated Polymer Gels for Mitigating Sediment Entrainment in Salt Caverns: A Robust Strategy for Safe CCUS Operations

**DOI:** 10.3390/gels12080732

**Published:** 2026-08-17

**Authors:** Erdong Yao, Kun Zhang

**Affiliations:** State Key Laboratory of Petroleum Resources and Engineering, China University of Petroleum (Beijing), Beijing 102249, China

**Keywords:** polymer gel, salt resistance, foam-templated consolidation, multiphase flow in porous media, sediment entrainment, CCUS underground infrastructure

## Abstract

As critical infrastructure for carbon capture, utilization, and storage (CCUS) and large-scale energy storage, subsurface salt caverns are seriously challenged by fluid-induced sediment mobilization during the supplementary debrining. Conventional bulk resin consolidation often causes severe viscous fingering, uneven consolidation, and pore clogging under hypersaline conditions. Here, we develop a foam-templated hybrid polymer gel co-stabilized by silica nanoparticles, polyvinyl alcohol, and the zwitterionic surfactant. The key novelty is the use of foam as a transient transport template that redistributes the resin phase and promotes selective cementation at grain-contact points instead of indiscriminate pore filling. This nano-reinforced gel system remained stable under hypersaline conditions (24% NaCl), and temperatures ranging from 20–80 °C. Micro-CT analysis showed that this selective templating preserved an interconnected pore network with a porosity above 45% and a CT-derived permeability of approximately 1.18 D, while reducing binder consumption by 55.6% relative to bulk resin injection. Crucially, a 1:200 geometrically scaled, velocity-matched pilot model demonstrated that this gel strategy limited sediment entrainment below 0.5% and reduced fluid discharge by 45.9%. These results establish a material-efficient consolidation strategy that combines sediment stabilization with permeability preservation, providing a promising solution for safer supplementary debrining in salt-cavern CCUS and energy-storage operations.

## 1. Introduction

**Figure 1:**
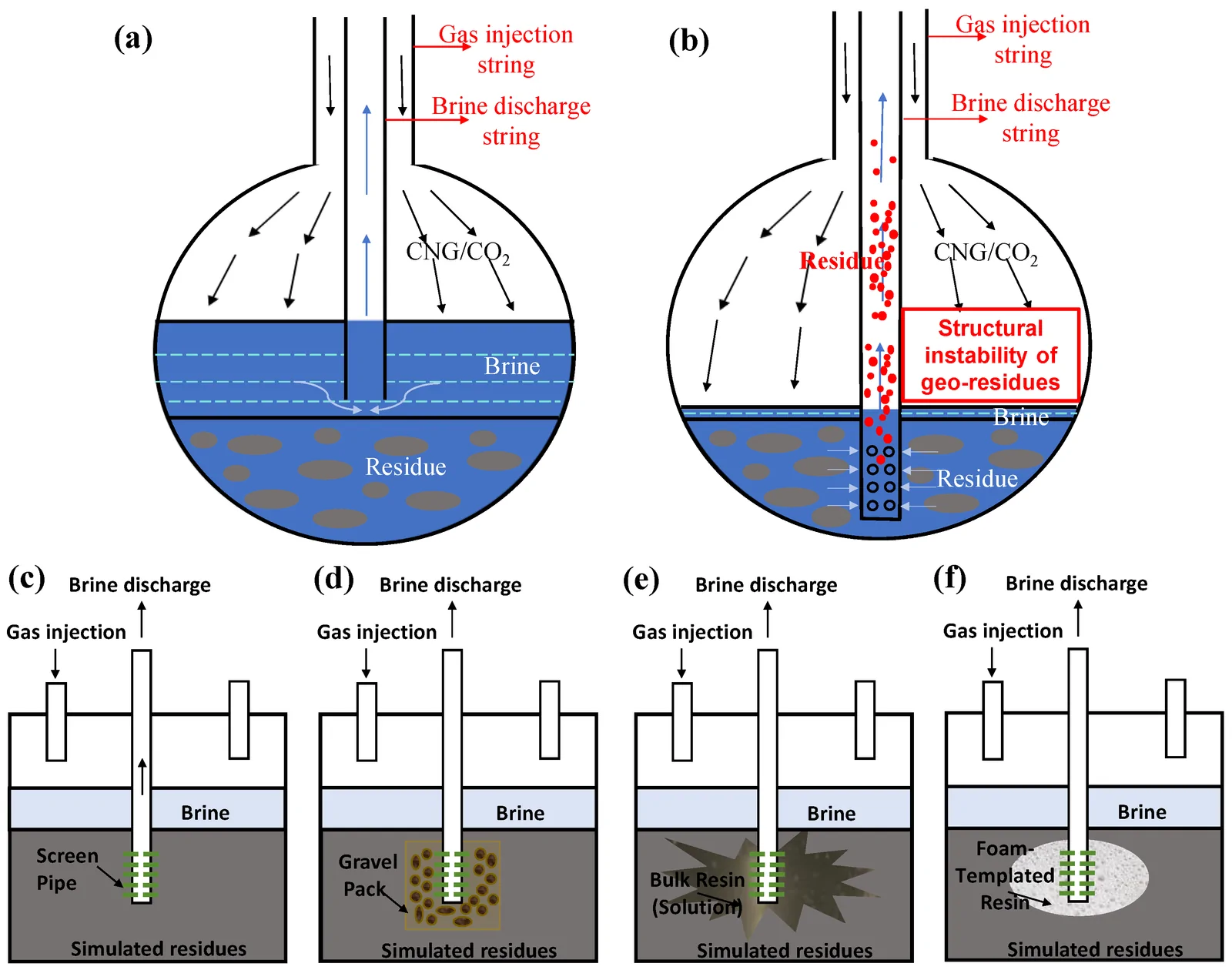
Schematic comparison of hydrodynamic stability and multiphase consolidation strategies during fluid discharge: (**a**) primary fluid displacement; (**b**) secondary displacement inducing structural instability in the granular matrix; (**c**) conventional mechanical retention; (**d**) irregular spatial distribution via bulk packing; (**e**) non-selective bulk resin injection causing severe pore clogging; and (**f**) foam-templated polymer gel structural stabilization.

The global transition toward carbon neutrality necessitates highly reliable subsurface infrastructure to support large-scale energy storage and advanced carbon management systems [1,2]. However, large-scale CCUS deployment still faces substantial challenges associated with technology readiness, infrastructure scale-up, and economic cost [3]. These constraints highlight the need for scalable and material-efficient technologies that can improve subsurface storage reliability while minimizing material consumption. In this context, salt cavern formations have emerged as premier geological storage vessels due to their exceptional sealing capacity, favorable geomechanical stability, and high injectivity [4]. These structural features render them highly effective for balancing temporal energy supply–demand dynamics [5]. Consequently, salt caverns are increasingly integrated into next-generation energy grids, serving as critical hosts for large-scale hydrogen containment, compressed air energy storage (CAES), and CO_2_ sequestration systems [3,6,7]. The fundamental transition of these caverns from initial solution mining to active energy storage relies entirely on primary debrining operations (Figure 1a) [8]. In low-impurity formations typical of Western regions [9], the operational risks during debrining can generally be mitigated through macroscopic flow regulation and optimized spatial well distribution [10,11,12].

Cavern geometry is also a critical design factor influencing storage capacity and operational stability. Previous shape-design studies have demonstrated that cavern dimensions and geometric configuration significantly affect deformation, volume convergence, and storage performance [5]. During debrining, such geometric heterogeneity may further complicate fluid distribution and hydrodynamic regulation, increasing the need for effective sediment stabilization to maximize usable cavern capacity. Furthermore, fluid flow disruptions induced by thermodynamic crystallization at elevated temperatures have been effectively managed via specialized backflushing fluid dynamics [13].

However, a highly complex operational challenge exists in high-impurity salt deposits, widely distributed in regions like China, where insoluble impurity fractions often exceed 50% [14]. Originating from continental sedimentary environments, these formations are rich in clastic rocks and clay minerals [15]. During the dissolution process, these insoluble phases accumulate at the cavern bottom, creating massive beds of unconsolidated granular residues [16,17,18]. To maximize the effective energy storage capacity, supplementary debrining (Figure 1b) is essential, theoretically reclaiming over 10% of the unused cavern volume [19,20]. Yet, the introduction of strong hydrodynamic forces during fluid withdrawal severely disrupts the static fluid-solid equilibrium, mobilizing fine particulates. This leads to massive sediment entrainment and catastrophic wellbore blockage, a fatal bottleneck that not only limits the ultimate storage capacity but also threatens the safety of long-term injection-withdrawal cycles [21,22,23].

Conventional particulate control strategies, such as mechanical screens (Figure 1c) and gravel packs (Figure 1d), lack the precise geometric adaptability required for these fine-grained, poly-disperse environments, and their deep-subsurface mechanical placement is often logistically unfeasible [24]. Chemical consolidation via reactive fluid injection offers superior adaptability [25]. However, field applications of conventional chemical consolidation remain challenged by permeability reduction and difficulty in selectively placing the consolidating agent within targeted formations [26,27]. Non-selective bulk resin injection may consequently occupy pore space and impair fluid conductivity (Figure 1e). Foam-templated polymer gels (Figure 1f) present a highly promising engineering alternative. Driven by hydrodynamic pressure gradients rather than requiring mechanical penetration into the residue, foam uniquely exploits the Jamin effect to generate localized capillary resistance, selectively retarding flow in high-permeability channels and drastically improving volumetric sweep efficiency [28]. Furthermore, utilizing the gas–liquid interface as a structural template enables directed polymer gel cementation specifically at grain-contact points, preserving critical pore connectivity while minimizing formation damage [29].

Recent studies have advanced foam-assisted flow control using branched preformed-particle-gel foams and pore-scale regulation of foam propagation under saline conditions [30,31]. Meanwhile, high-temperature and salt-resistant polymer gels have achieved strong bulk plugging performance in heterogeneous reservoirs [32]. Resin-foam systems have improved sand consolidation while reducing formation damage, whereas advanced foams and polymer gels have mainly enhanced mobility control, conformance, and plugging performance. In contrast, the present strategy employs foam as a transient sacrificial transport template to redistribute epoxy resin toward grain-contact regions, thereby combining permanent sediment consolidation with pore-connectivity preservation and reduced binder consumption.

In this study, we propose a foam-templated polymer gel stabilization strategy to mitigate fluid-induced sediment entrainment during the critical capacity-reclamation phase of subsurface energy storage. This work systematically engineers a nano-reinforced hybrid polymer gel capable of exhibiting outstanding thermal and salinity tolerance under extreme hypersaline (24% NaCl) and elevated temperature (20–80 °C) conditions. Furthermore, we elucidate its multiphase transport mechanisms within unconsolidated media and validate its macroscopic engineering efficacy through large-scale (1:200) physical simulations scaled directly from actual field salt cavern hydrodynamics. By resolving this critical flow bottleneck, this study provides a scalable, high-performance polymer gel engineering solution to enhance operational safety, maximize reclaimable capacity, and extend the lifecycle of global subsurface energy and CCUS infrastructure.

## 2. Results and Discussion

### 2.1. Geological and Granulometric Characteristics of Cavern Residues

**Figure 2:**
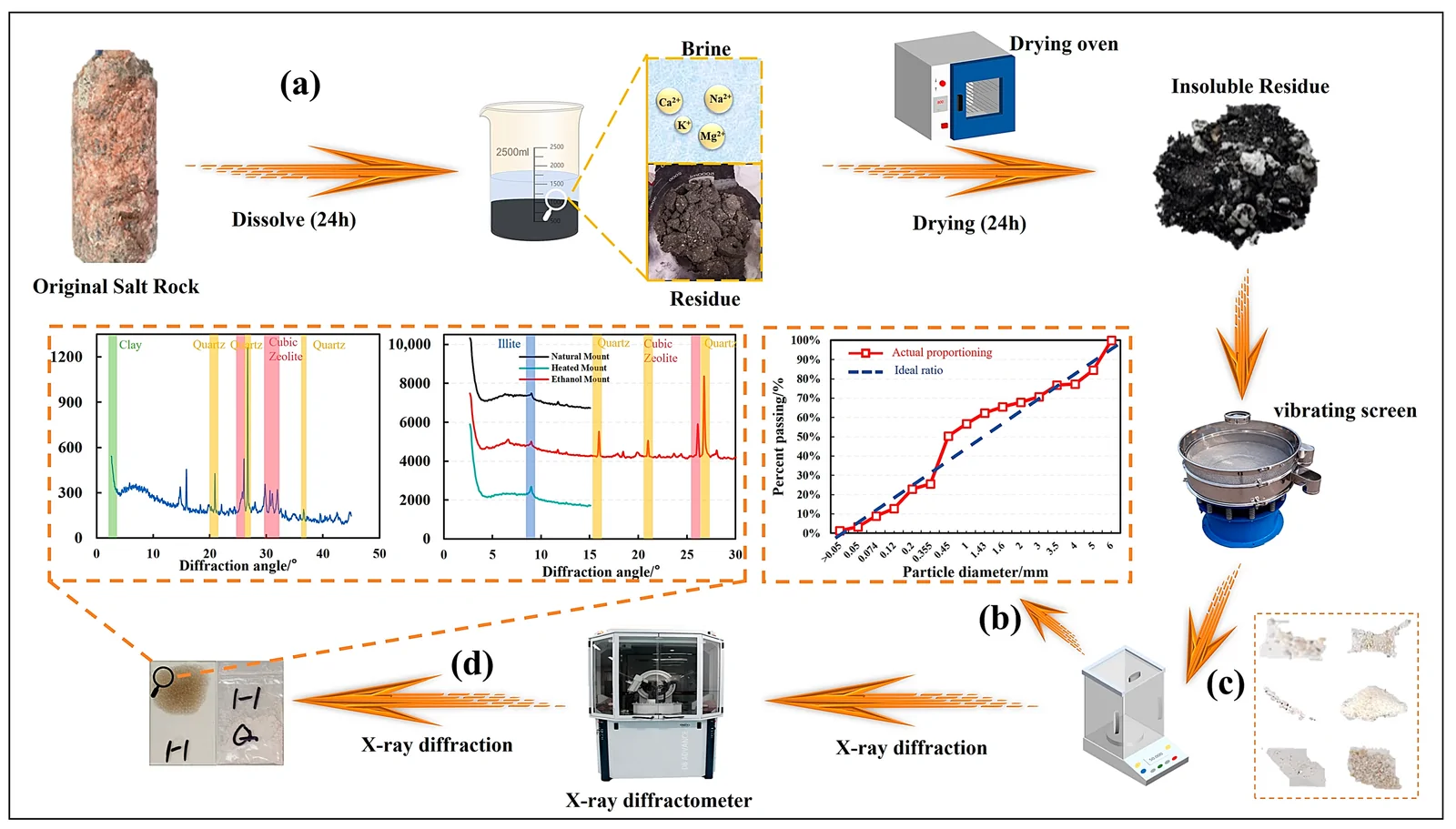
Systematic physicochemical and granulometric characterization workflow for reconstructing the porous matrix: (**a**) recovery of insoluble particulate phases via controlled dissolution; (**b**) granulometric profiling via mechanical sieving; (**c**) formulation of the standardized quartz porous matrix based on the in situ particle size distribution (PSD); and (**d**) mineralogical quantification via X-ray diffraction (XRD).

To establish a highly reproducible and representative engineering baseline, a comprehensive characterization of the native unconsolidated granular residues retrieved from an operational salt cavern gas storage facility was conducted. The workflow encompassed systematic field sampling, mineralogical quantification, and granulometric evaluation (Figure 2).

Quartz was identified as the predominant insoluble phase (27.1 wt%), complemented by cubic zeolite (21.5 wt%) and a total clay colloidal fraction of 15.6 wt% (with a negligible montmorillonite content of 0.6 wt%).

Mechanical sieving analysis (using 4, 7, 10, 20, 100, and 200 mesh standards) revealed a poorly sorted, polymodal particle size distribution (PSD). The native particulate matrix was characterized by three distinct dominant fractions: 3.5–6 mm (4–7 mesh), 1–2 mm (10–20 mesh), and 0.074–0.154 mm (100–200 mesh) [23,29].

To replicate the in situ multi-scale pore network and packing density, a heterogeneous blend was formulated at a 4:4:2 mass ratio across these three dominant particle-size fractions. The mineralogical composition of the reconstructed matrix was separately adjusted according to the semi-quantitative XRD results. This optimized matrix provides the physical foundation for the subsequent foam-templated polymer gel stabilization studies.

### 2.2. Formulation and Hypersaline Robustness of the Foam-Templated Gel

#### 2.2.1. Screening of the Continuous Polymeric Binder

The high-shear dispersion results (Table 1) indicate that while all four polymer-surfactant systems successfully generated a macro-emulsion foam, their drainage kinetics differed among the formulations.

Quantitative analysis of the F_c_ values reveals that the epoxy and urea-formaldehyde colloidal systems exhibited superior comprehensive stability. Although the urea-formaldehyde system demonstrated a high absolute volume, its resulting cross-linked network lacks the mechanical robustness and long-term hydrolytic stability required for extreme subsurface environments. In contrast, the epoxy-based multiphase system exhibited an exceptional half-life (99.5 min), indicating superior viscoelasticity in the lamellae, effectively retarding film drainage. Coupled with its robust cross-linking density, high-temperature tolerance, and favorable interfacial compatibility [33], the epoxy resin was strategically selected as the foundational continuous phase for subsequent interfacial optimization and multiphase flow studies.

It should be noted that this initial fluid screening was designed as a targeted engineering pre-evaluation. This initial resin screening was conducted as a preliminary engineering comparison. Independent replicate measurements were not performed; therefore, standard deviations, confidence intervals, and statistical significance tests are not available for these data. All formulations were evaluated under identical calibrated isothermal conditions to maintain consistent comparative conditions.

#### 2.2.2. Component Optimization Under Hypersaline Stress

The operational success of a stabilization fluid in CCUS and CAES salt caverns dictates that it must withstand aggressive geochemical conditions—specifically, extreme hypersalinity and elevated temperatures—without premature structural collapse. To achieve this, the chemical composition of the multiphase system was systematically screened to establish a functional formulation for subsurface conditions.

(1) Securing Interfacial Stability Under Hypersaline Stress:

The generation of a stable gas–liquid interface requires a surfactant capable of withstanding the severe electrical double-layer compression induced by saturated formation brines. We systematically evaluated a series of mobility control agents (Tween 80, SDS, BS-12, and LHSB) [33]. Under saturated brine conditions (simulating the extreme 24% NaCl salinity of storage caverns), only the zwitterionic surfactant dodecyl dimethylamine ethyl lactone (BS-12) maintained exceptional stability.

**Figure 3:**
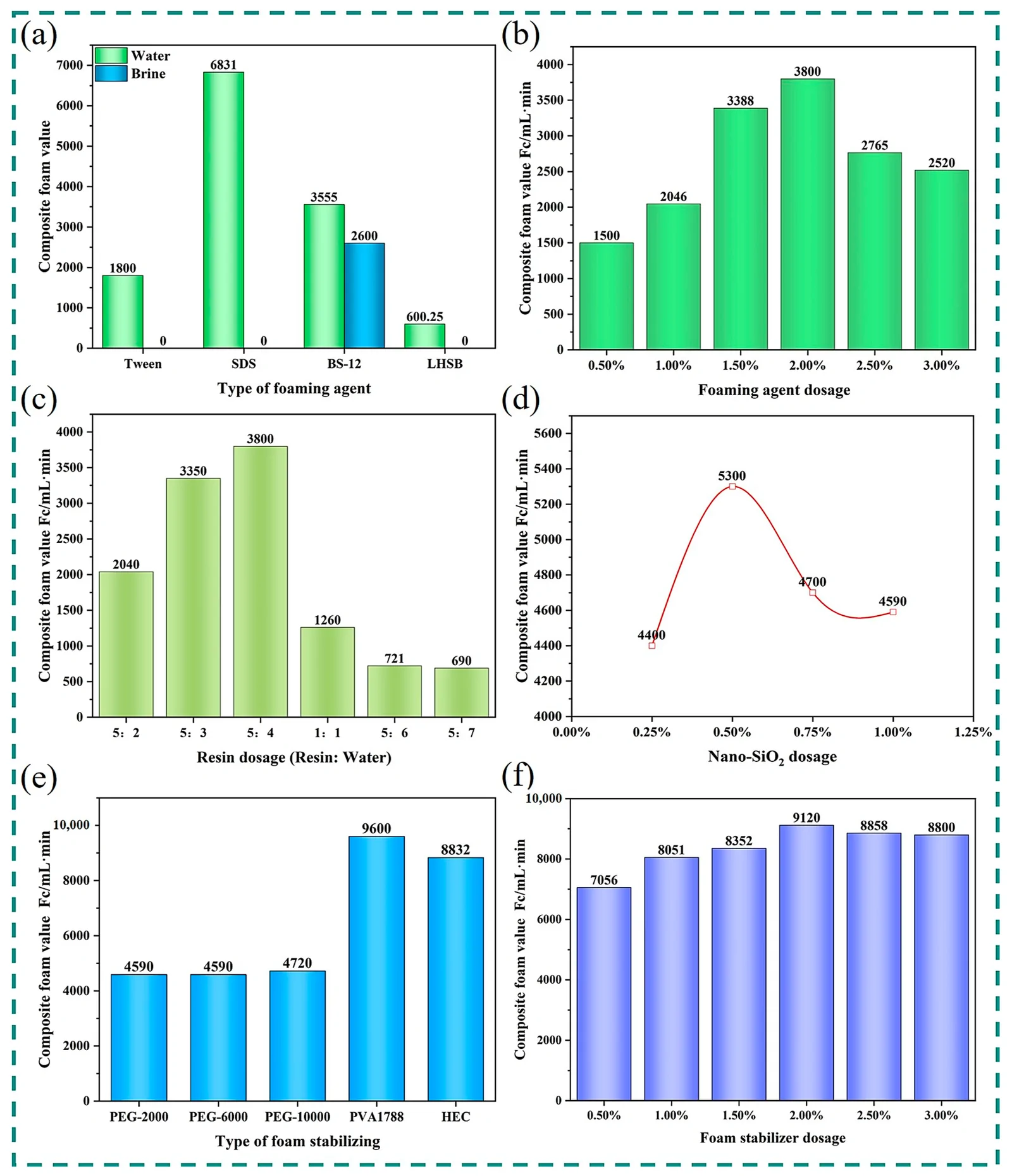
Interfacial assembly and component optimization of the multiphase colloidal system: (**a**) screening of amphiphilic surfactants; (**b**) optimization of surfactant (BS-12) concentration; (**c**) determination of the dispersed-to-continuous phase volume ratio; (**d**) concentration optimization of the nanoparticle stabilizer (nano-SiO_2_); (**e**) evaluation of synergistic polymer co-stabilizers; and (**f**) synergistic enhancement of the comprehensive colloidal index.

Unlike conventional anionic surfactants (like SDS, which suffered immediate double-layer collapse and a stability index of 0 mL·min), the zwitterionic molecular architecture of BS-12 provides internal charge compensation. This forms a localized ‘ion shielding layer’ that drastically reduces the electrostatic interference of massive Na^+^ counterion concentrations on the interfacial film. Consequently, its comprehensive stability index reached 2600 mL·min (Figure 3a). Concentration optimization revealed a peak performance at a BS-12 dosage of 2.0% (Figure 3b), establishing it as the primary agent for ensuring fluid longevity in hypersaline caverns.

(2) Rheological Optimization for Multiphase Injection:

The volume ratio between the dispersed structural binder (resin) and the continuous aqueous phase (brine) critically governs the system’s apparent viscosity and injectivity. Ratios exceeding 5:3 yielded excessively high fluid viscosity, which would severely hinder deep matrix penetration during field injection and require impractically high pumping pressures. Conversely, excessively increasing the aqueous phase compromised the structural longevity of the fluid template. Through rigorous hydrodynamic assessments, an optimal phase volume ratio of 5:4 was determined (Figure 3c), striking a precise engineering balance between favorable injection mobility and the necessary structural rigidity of the foam template [34].

(3) Nanoparticle-Polymer Synergy for Long-Term Stabilization:

To further mitigate fluid drainage under the high-pressure gradients of cavern operations, a dual-stabilization strategy was engineered. Initially, hydrophilic silica nanoparticles (nano-SiO_2_) were introduced at an optimal dosage of 0.5% (Figure 3d). These nanoparticles irreversibly adsorb at the gas–liquid interface, constructing a rigid mechanical barrier that physically arrests film drainage.

Subsequently, the synergistic interaction between this particulate barrier and various water-soluble polymeric networks was evaluated to enhance the viscoelasticity of the continuous phase. Polyvinyl alcohol (PVA1788) emerged as the superior co-stabilizer. The integration of 0.5% nano-SiO_2_ and 2.0% PVA1788 synergistically increased the comprehensive stability index to 9120 mL·min—a 30.1% enhancement over using the polymer alone (Figure 3e,f). Microscopically, this enhancement may be associated with possible hydrogen-bonding interactions [35]. This engineered synergy ensures the multiphase fluid maintains a robust, highly stable structural template, providing the critical temporal window necessary for the resin to penetrate the granular matrix and initiate cross-linking during subterranean deployment.

It is important to note that the current component optimization was conducted using a single-variable approach to rapidly establish a functional engineering baseline for the subsequent pilot-scale simulations. While the selected formulation demonstrated satisfactory performance in the subsequent pilot-scale validation, the present single-variable approach does not establish a global optimum. Future studies will employ multivariable experimental designs, such as Response Surface Methodology, to systematically evaluate component interactions and further optimize the formulation.

#### 2.2.3. Thermal Robustness and Salinity Tolerance

Subsurface environments impose severe thermodynamic and physicochemical stresses on colloidal fluids. We evaluated the structural resilience of the optimized nanoparticle-polymer foam system across varying salinities and temperatures.

(1) Salinity Tolerance Mechanisms

**Figure 4:**
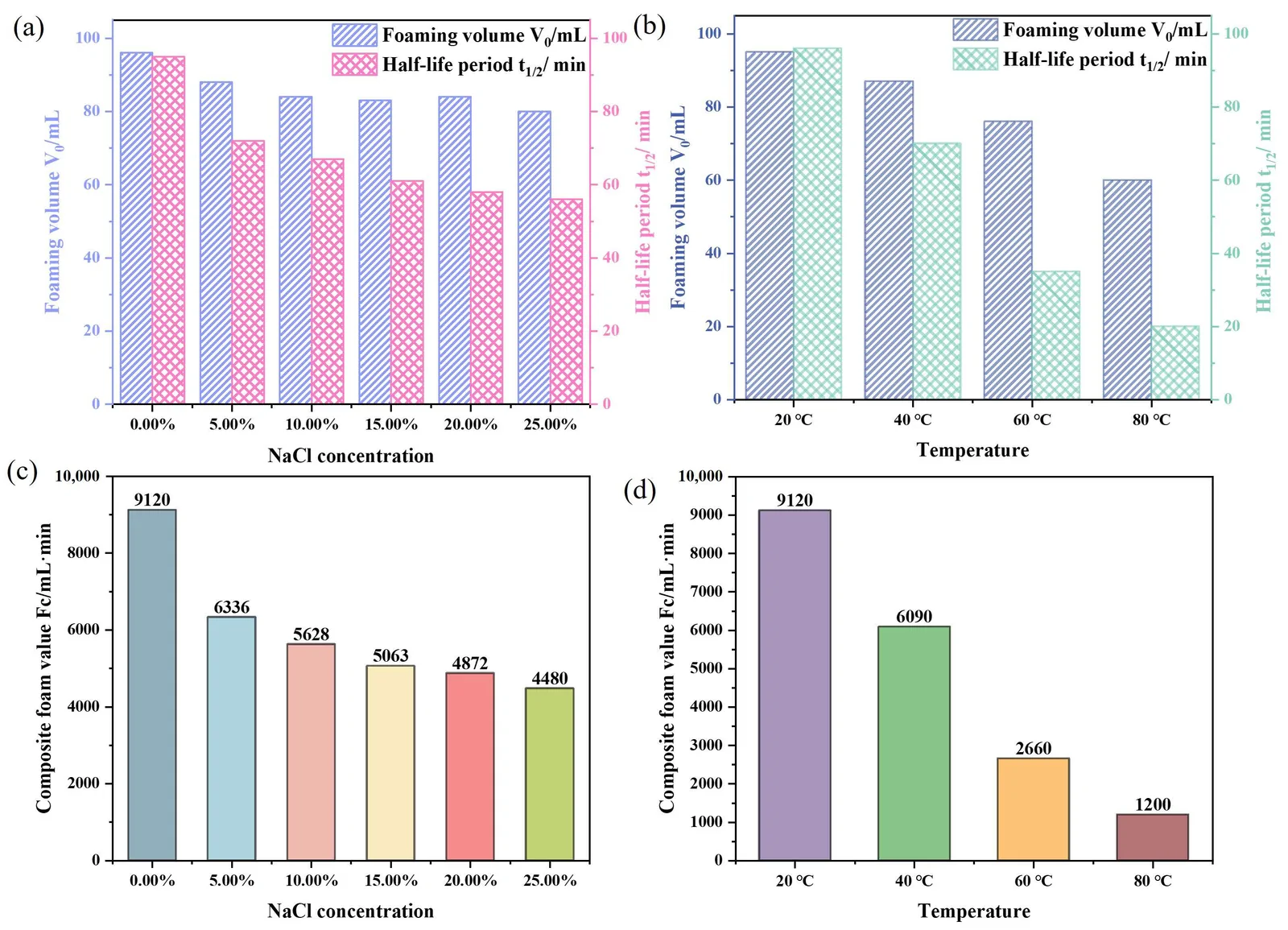
Thermodynamic and physicochemical robustness of the nanoparticle-polymer enhanced foam: (**a**) impact of ionic strength (NaCl concentration) on interfacial generation and drainage time; (**b**) thermal stability profile; (**c**) comprehensive colloidal stability under hypersaline conditions; (**d**) colloidal longevity under elevated thermal stress.

To simulate dynamic in situ formation water, the complex fluid was exposed to varied NaCl concentrations (Figure 4a,c). While elevated ionic strength moderately reduced the initial interfacial generation volume (maintained >55 mL with no phase separation), the drainage half-life remained astonishingly robust (consistently >80 min). Unlike conventional anionic lamellae that undergo head-group dehydration and rapid coalescence under hypersaline conditions (e.g., 24% NaCl), the isoelectric point adjustment provided by the betaine structure (BS-12) sustains a highly cohesive interfacial film, confirming excellent salt tolerance [36].

(2) Thermal Stability and Drainage Kinetics

The colloidal stability was evaluated over a critical temperature spectrum of 20–80 °C (Figure 4b,d). Elevated thermal energy inherently accelerates the evaporation of the aqueous phase within the lamellae and reduces the bulk viscosity, intensifying Plateau border drainage [37]. Consequently, the half-life decreased from 70 min at 40 °C to approximately 20 min at 80 °C. In practical deployment, however, the formulation is initially injected near ambient temperature and progressively warms during subsurface transport. The longer foam stability and slower curing at the initial lower temperature favor foam propagation and resin redistribution, whereas cross-linking accelerates as the system approaches the formation temperature [38]. Therefore, the ~20 min half-life at 80 °C represents the residual foam stability at the highest tested isothermal temperature rather than the total available placement and curing time.

#### 2.2.4. Curing-Agent Selection and Morphological Fixation

The transition from a dynamic foam template to a consolidated porous skeleton benefit from delayed curing during fluid placement, followed by effective cross-linking after resin redistribution. In practical deployment, the formulation is initially injected at near-ambient temperature and gradually heated toward the formation temperature, which helps suppress premature curing during transport while promoting faster cross-linking after placement [39].

Three candidate curing agents were evaluated: polyetheramine D230 [40] and phenolic amines NX-2003 and NX-2003D [41]. The multiphase system, with a resin-to-curing-agent ratio of 3:1, was mixed with unconsolidated quartz sand (20–40 mesh) to evaluate its macroscopic setting and structural fixation behavior. For D230, an E51/D230 mass ratio of 3:1 was adopted as a near-stoichiometric formulation. Based on the 1:1 stoichiometric balance between epoxy equivalents and active amine-hydrogen equivalents, an E51 epoxy equivalent weight of 184–194 g·eq^−1^ and a D230 amine-hydrogen equivalent weight of approximately 60 g·eq^−1^ give a theoretical mass ratio of 3.07–3.23:1, close to the adopted 3:1 ratio and consistent with reported DGEBA/D230 curing systems [39].

**Figure 5:**
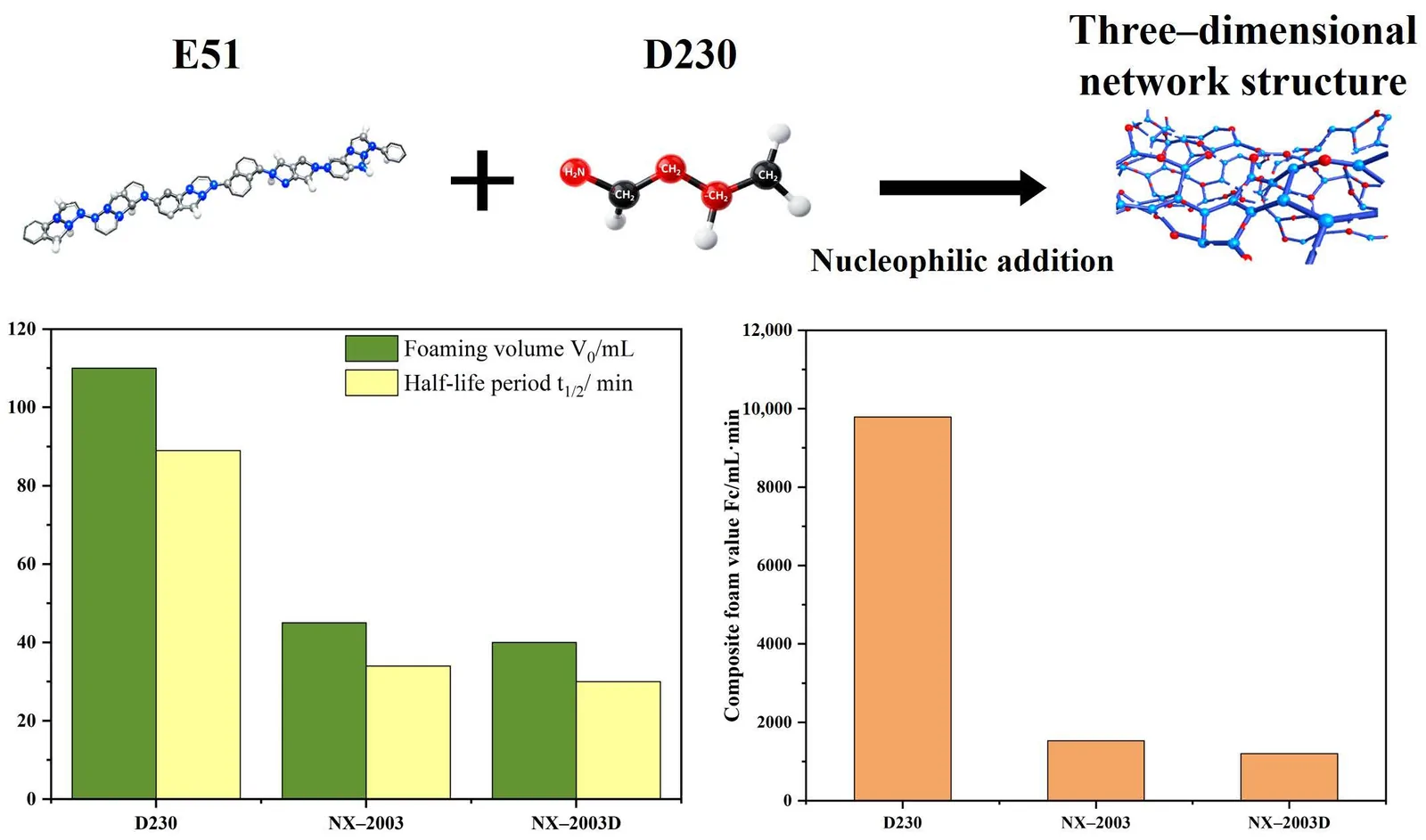
Effect of different curing agents on foam stability and qualitative curing-agent selection.

Among the tested curing agents, D230 exhibited the most favorable setting performance, forming a transparent and resilient polymer network with reported thermal stability from −35 °C to 120 °C [42]. As shown in Figure 5, D230 effectively fixed the foam-templated morphology after resin placement, enabling effective consolidation with reduced polymer consumption. However, these observations provide qualitative rather than quantitative evidence of structural fixation.

#### 2.2.5. Microstructural Architecture and Interfacial Networking of the Cured Gel

**Figure 6:**
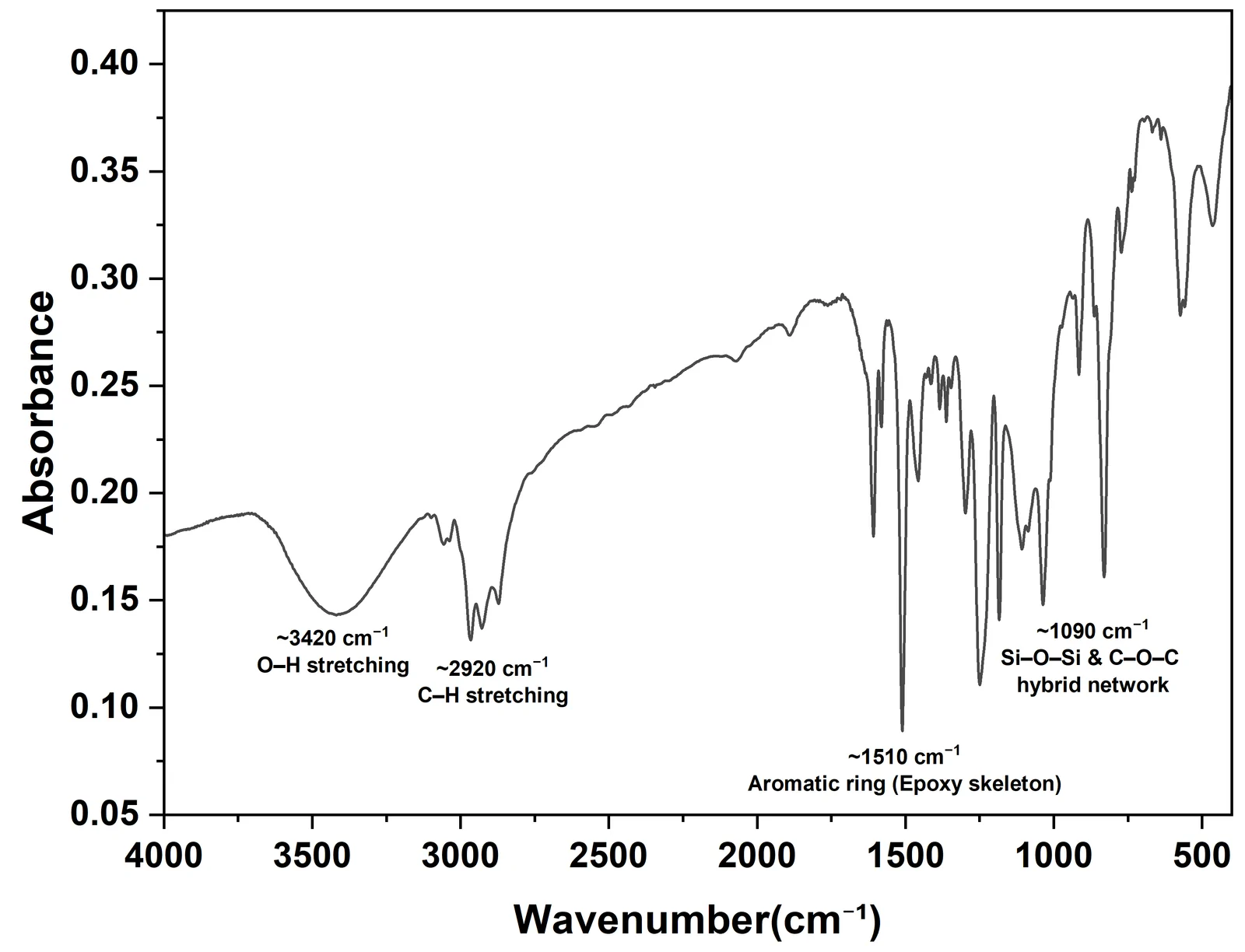
FTIR spectrum showing characteristic spectral features of the cured hybrid gel matrix.

To elucidate the chemical structure and cross-linking mechanism of the foam-templated matrix, FTIR spectroscopy was performed on the cured composite (Figure 6). The characteristic absorption band at ~3420 cm^−1^ is assigned to O–H stretching vibrations, suggesting possible hydrogen-bonding interactions between the polyvinyl alcohol (PVA) co-stabilizer and the hydroxyl groups generated during epoxide ring-opening of the E51 resin. The sharp doublet peaks at ~2920 cm^−1^ and ~2850 cm^−1^ correspond to asymmetric and symmetric C–H stretching vibrations of aliphatic chains derived from the zwitterionic surfactant (BS-12) and the polymer backbone. Crucially, the prominent absorption peak at ~1510 cm^−1^ represents the aromatic C=C skeletal vibration of the bisphenol-A epoxy resin, confirming the presence of the aromatic epoxy framework within the cured matrix. Furthermore, the broad, intense absorption band spanning ~1080–1100 cm^−1^ is attributed to the overlapping vibrations of Si–O–Si and C–O–C ether linkages. This is consistent with an organic–inorganic hybrid network containing hydrophilic nano-SiO_2_ within the polymeric matrix. However, FTIR alone cannot distinguish chemical bonding from physical entrapment or directly quantify the resulting interfacial reinforcement.

**Figure 7:**
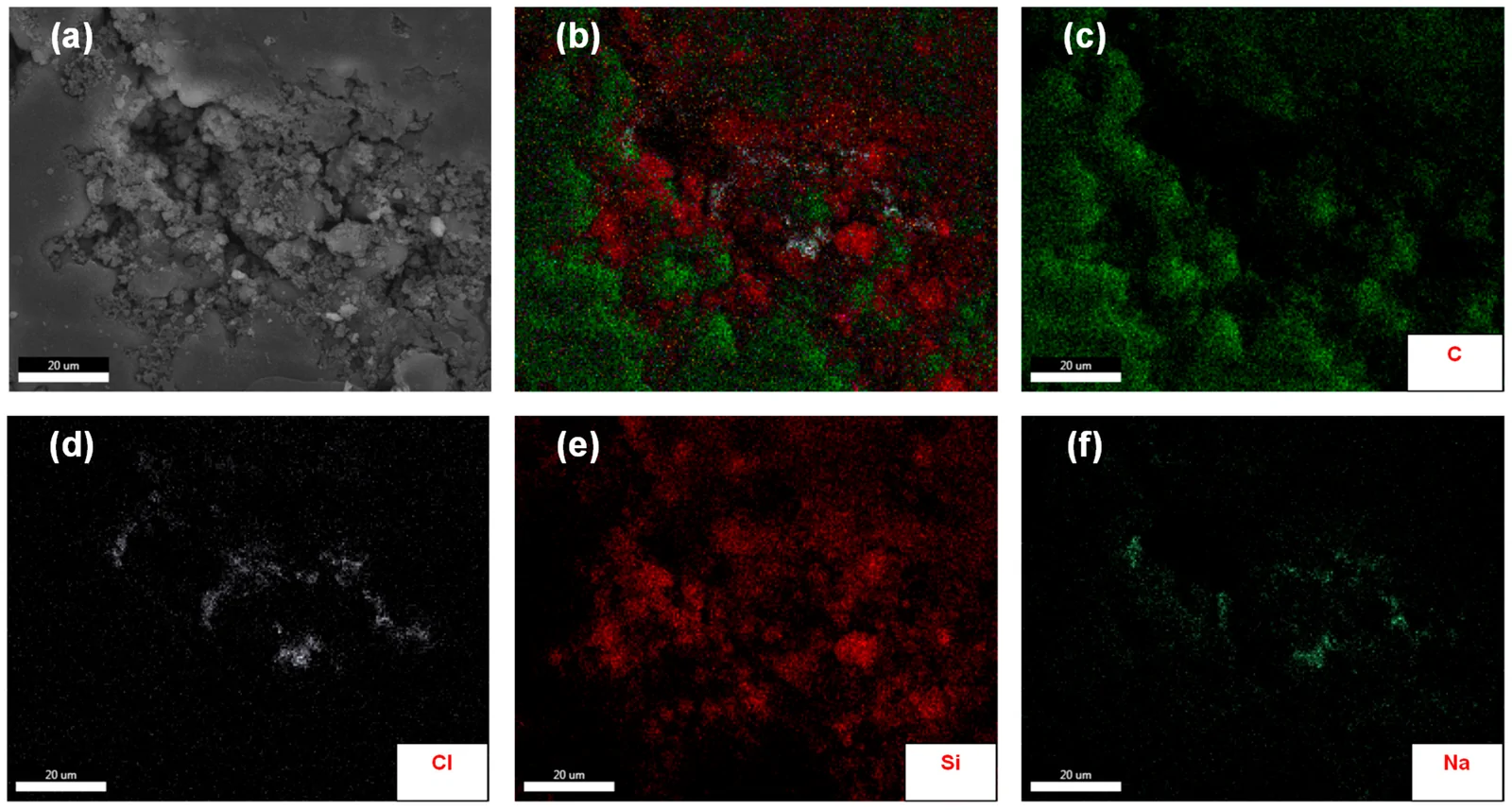
SEM micrograph (**a**) and corresponding EDS mappings (**b**–**f**) of the cured polymer gel matrix: (**b**) all-element overlay; (**c**) C (polymer framework); (**d**) Cl and (**e**) Si (nano-SiO_2_-associated signal/distribution); (**f**) Na (salinity background).

To further evaluate the structural uniformity and environmental robustness of this cross-linked network under hypersaline subterranean conditions, SEM coupled with EDS elemental mapping was conducted on the cured matrix (Figure 7). As illustrated in Figure 7a, the cured gel exhibits a well-developed, continuous three-dimensional porous architecture without lamellar collapse or phase separation, even when cured under saturated brine (24% NaCl) conditions. The corresponding all-element overlay (Figure 7b) and individual elemental mappings confirm that the polymeric carbon framework (Figure 7c, C) is homogeneously distributed across the entire cellular skeleton.

The detected distributions of Cl and Na reflect the presence of the hypersaline environment within the cured matrix. The relatively uniform Si distribution is consistent with the incorporation and dispersion of nano-SiO_2_ within the polymer matrix. Together, these results confirm the coexistence and spatial distribution of the principal organic and inorganic components within the cured matrix.

### 2.3. Mechanical Consolidation and Pore-Structure Preservation

#### 2.3.1. Mechanical Consolidation and Geomechanical Performance

**Figure 8:**
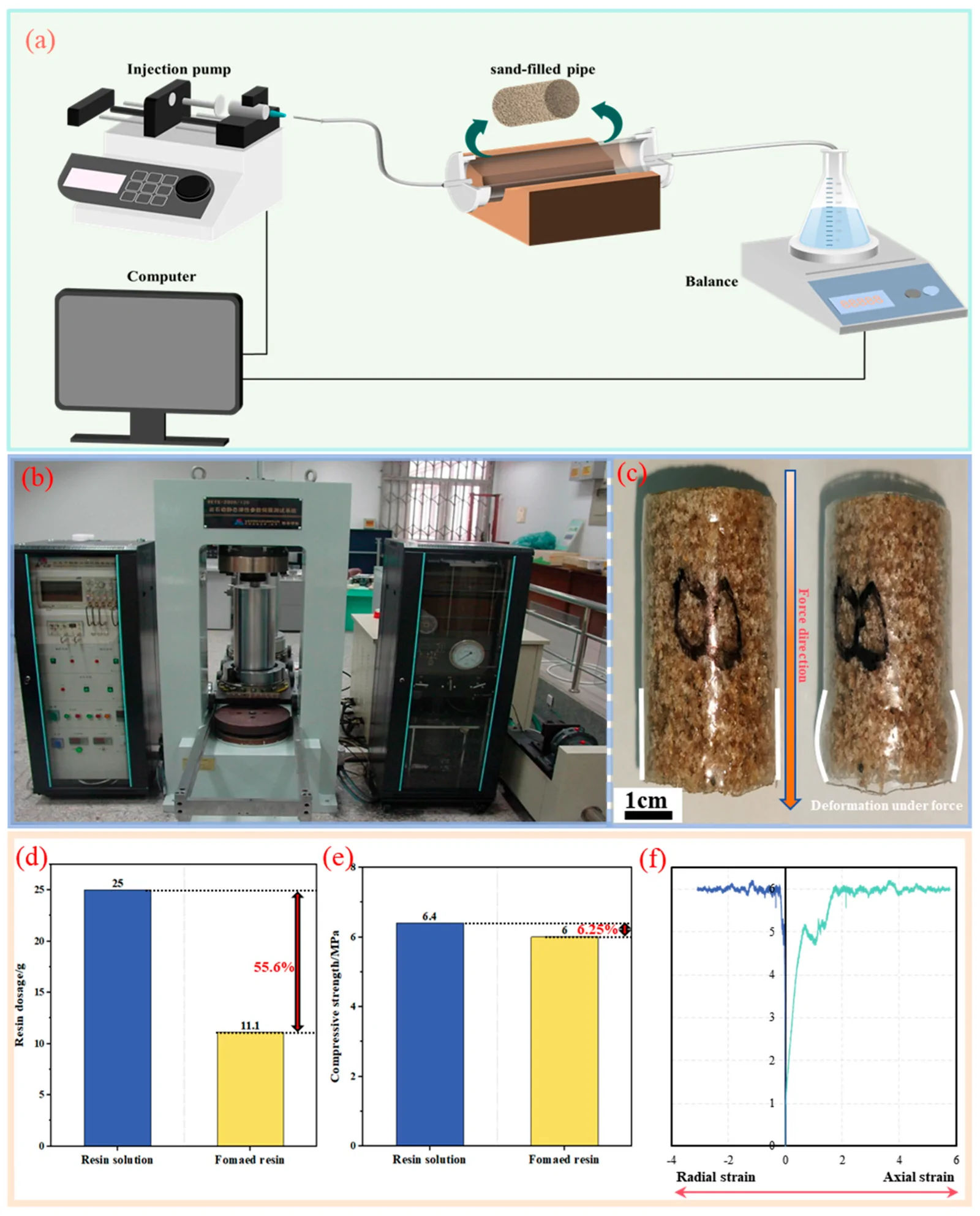
Multiphase flow simulation and macroscopic geomechanical evaluation: (**a**) process flow schematic of the core-flooding apparatus; (**b**) detailed configuration of the multiphase injection system; (**c**) morphological fixation of the consolidated matrices; (**d**) foam-templated reduction in continuous phase (resin) consumption; (**e**) comparative geomechanical strength; and (**f**) stress–strain profiling of the structurally stabilized matrix.

The foam-templated gel mechanism drastically optimized the material efficiency. By utilizing the gas–liquid interface to direct the polymer placement, the foam system reduced the required dispersed resin mass by 55.6% compared to the bulk continuous fluid injection (Figure 8d). Despite this significant reduction in chemical dosage, the consolidated gel matrix exhibited robust geomechanical stability, maintaining a characteristic peak compressive stress of approximately 6 MPa (Figure 8c–f). Together with the post-failure core morphology, these results indicate that effective mechanical consolidation can be achieved through targeted polymer gel cementation despite the reduced resin dosage.

#### 2.3.2. Micro-CT Tomography and Hydrodynamic Conductivity Preservation

**Figure 9:**
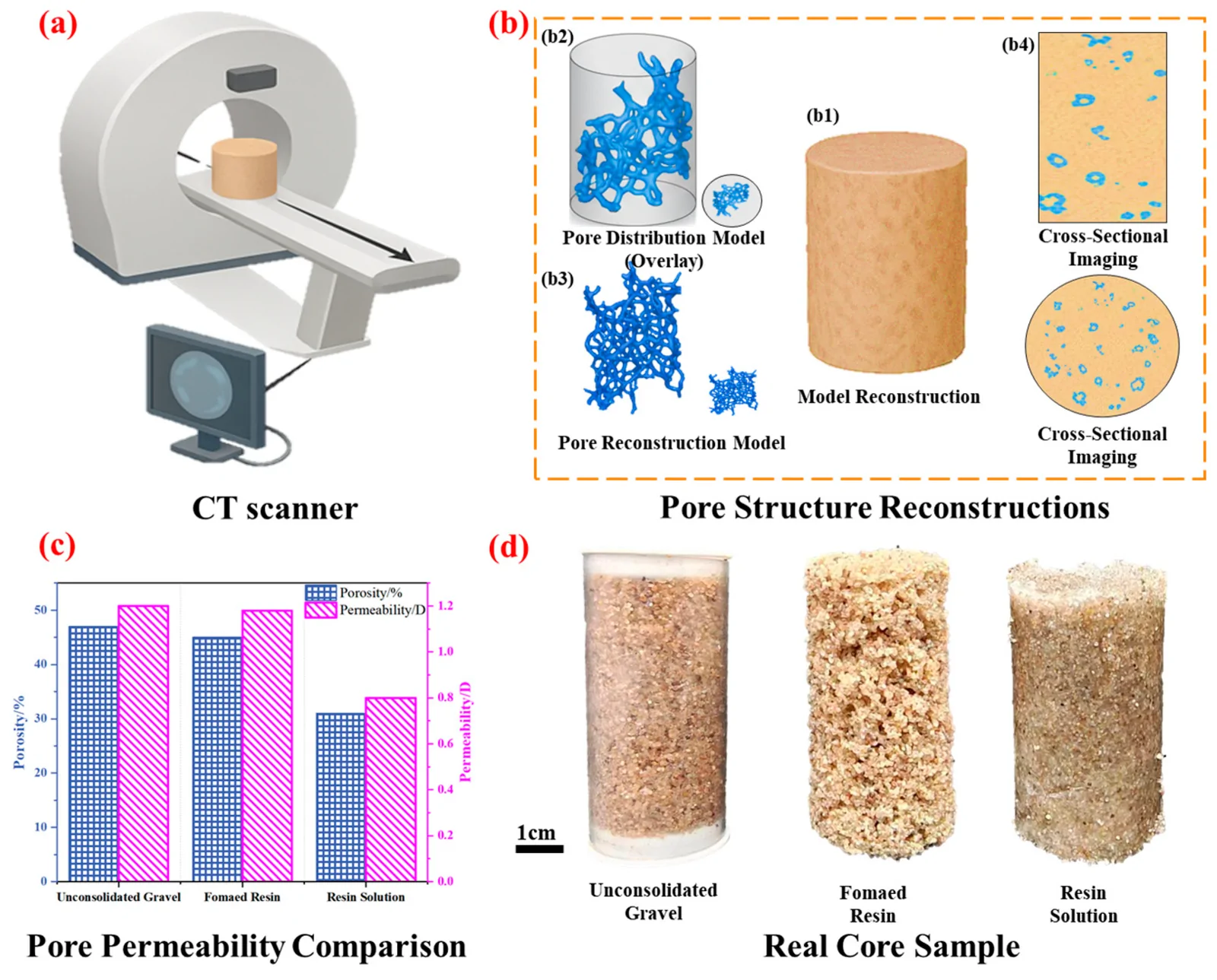
Petrophysical and tomographic characterization of the stabilized porous media: (**a**) high-resolution Micro-CT scanning; (**b**) 3D pore network reconstructions; (**c**) comparative evaluation of preserved porosity and hydrodynamic conductivity (permeability); (**d**) macroscopic morphology of the consolidated matrix.

**Figure 10:**
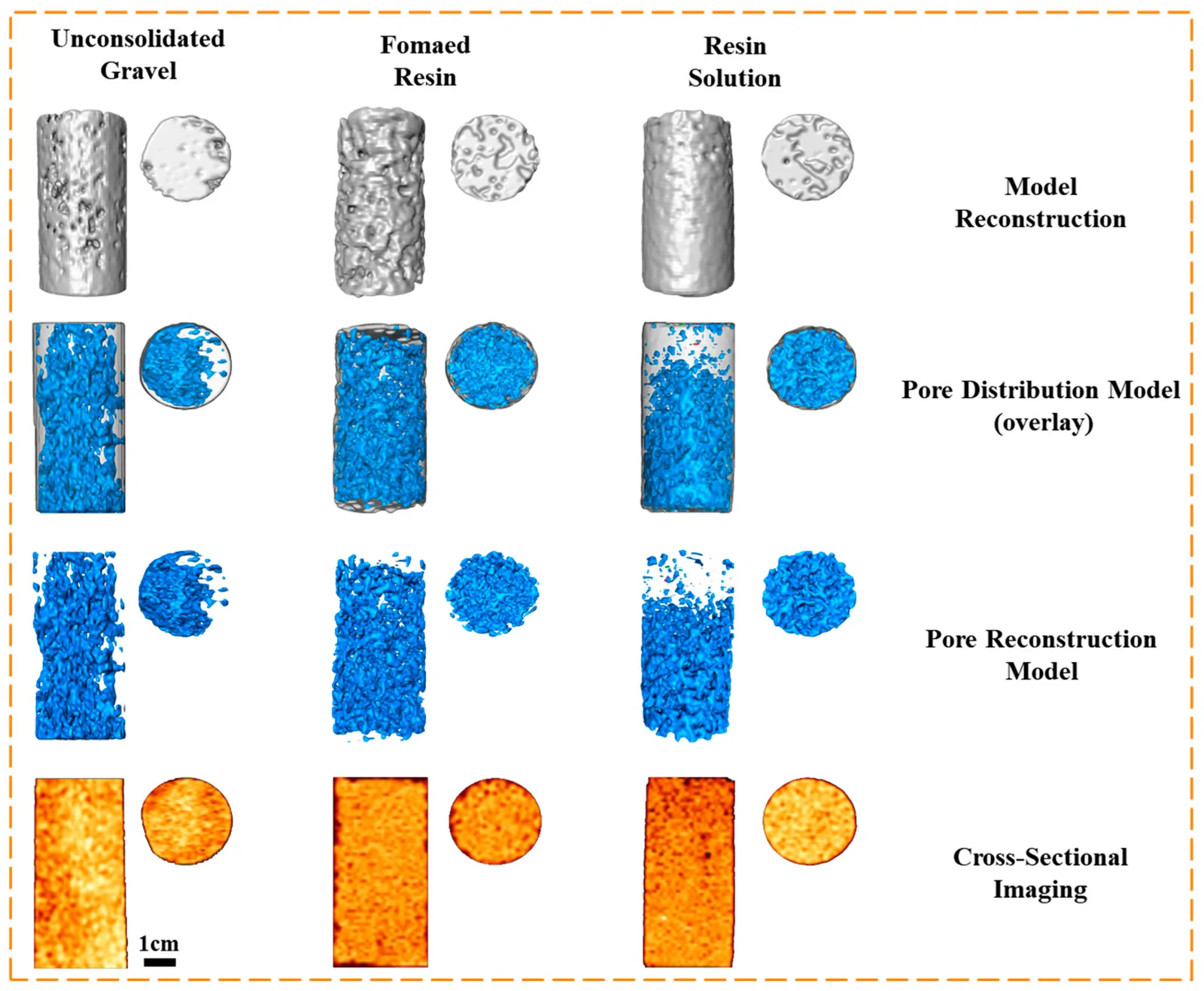
Comparative micro-tomographic analysis of hydrodynamic conductivity and interstitial void preservation among the unconsolidated matrix, foam-templated polymer gel system, and bulk resin solution.

(1) Bulk Single-Phase Fluid Dynamics (Control): The conventional resin solution, driven by highly unfavorable mobility ratios, exhibited severe flow heterogeneity. Micro-CT reconstruction (Figure 9 and Figure 10) revealed a high initial consolidation density that rapidly diminished distally. This Newtonian fluid completely occupied the interstitial voids in the entry regions, leading to severe localized pore clogging. Consequently, the bulk porosity collapsed to approximately 30%, and CT-derived permeability estimate of approximately 0.8 D.
(2) Interfacial-Directed Foam Templating: Conversely, the foam-templated system demonstrated vastly superior, homogeneous spatial distribution. Guided by localized capillary forces and the Jamin effect, the foam selectively navigated the complex pore architecture. Upon rupture and regeneration, the liquid lamellae directed the reactive resin specifically to grain contact points—forming load-bearing pendular rings—rather than flooding the pore bodies.
(3) Hydrodynamic Preservation: As validated by the Micro-CT 3D pore network models, the foamed gel critically preserved a highly interconnected void structure. The porosity was remarkably maintained at approximately 45% (a 50% relative increase over the bulk resin), and the hydrodynamic conductivity reached 1.18 D (a 47.5% enhancement).

By shifting the paradigm from “bulk fluid filling” to “foam-templated gel templating,” this strategy achieves high-strength consolidation while effectively mitigating the fluid dynamic bottlenecks inherent in conventional stabilization techniques.

### 2.4. Multiphase Transport and Interfacial Cementation Mechanisms

#### 2.4.1. Interfacial Pendular Bridging vs. Bulk Pore Clogging

**Figure 11:**
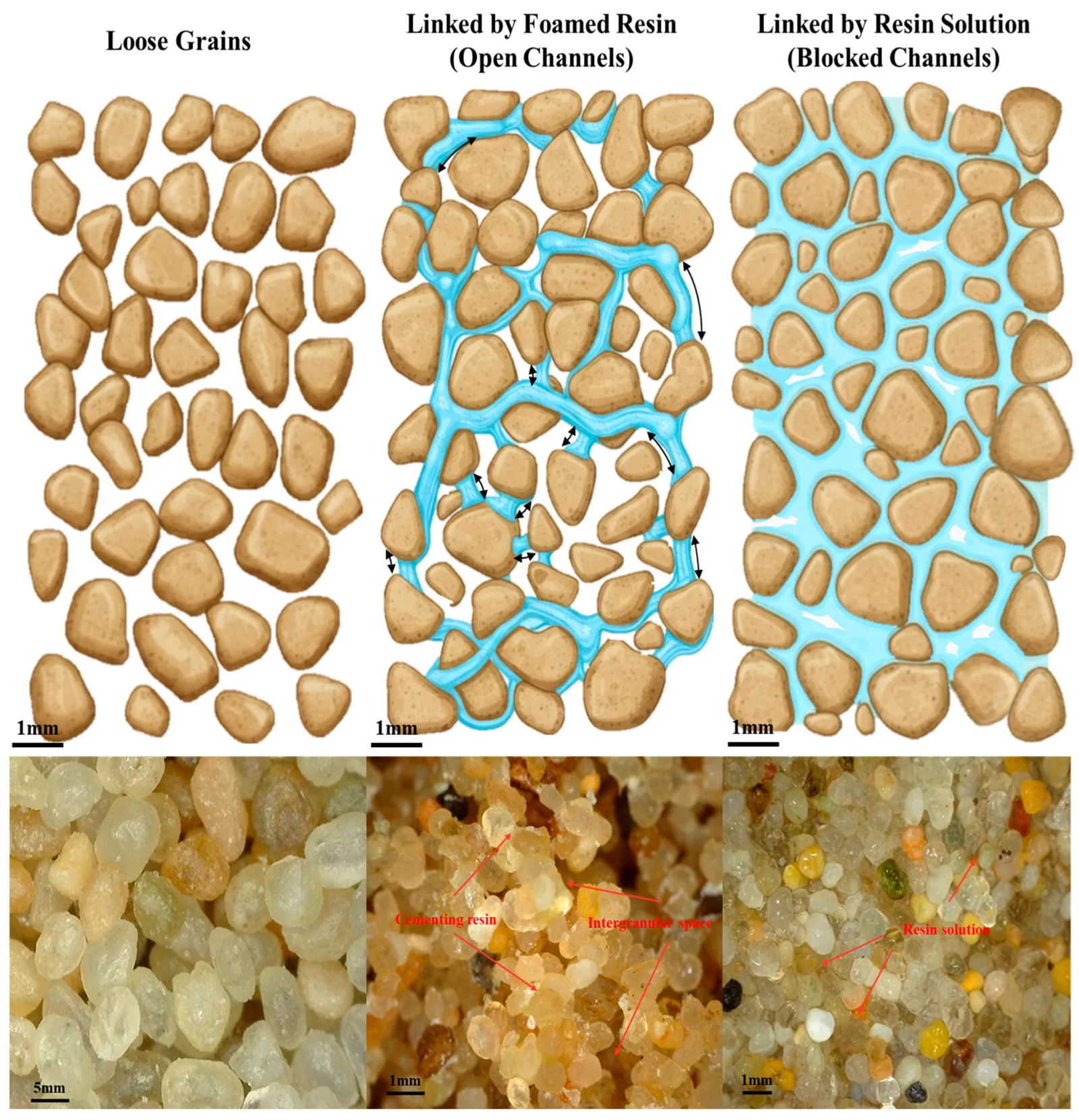
Microscopic visualization of polymer gel cementation versus bulk fluid clogging: (**Left**) Unconsolidated baseline matrix; (**Middle**) foam-templated gel bridging showing robust pendular rings and preserved voids; and (**Right**) bulk single-phase resin demonstrating severe localized pore clogging.

Compared to conventional bulk injection, the foam-templated system achieves robust mechanical consolidation while drastically reducing polymer consumption. Microscopic analysis of the consolidated matrices (Figure 11) reveals a fundamental paradigm shift in polymer gel cementation:

Interfacial-Directed Templating: In the foam-templated core, the reactive polymer gel is selectively distributed at the solid–liquid interface, adsorbing uniformly onto quartz surfaces to form load-bearing pendular rings at grain junctions. This preserves interstitial connectivity, maintaining pore throat diameters of 1–2 mm. The dispersed gaseous phase acts as a dynamic structural template, directing the gel binder to contact points while keeping the pore bodies open.

Bulk Fluid Clogging: Conversely, bulk resin solution indiscriminately fills the interstitial volume, causing catastrophic pore throat occlusion (pores < 0.3 mm). While this increases localized compressive strength, it obliterates hydrodynamic conductivity. This failure is driven by the extreme Newtonian viscosity and unfavorable mobility ratio, leading to preferential channeling and permanent entrapment of unreacted resin in smaller capillaries.

**Figure 12:**
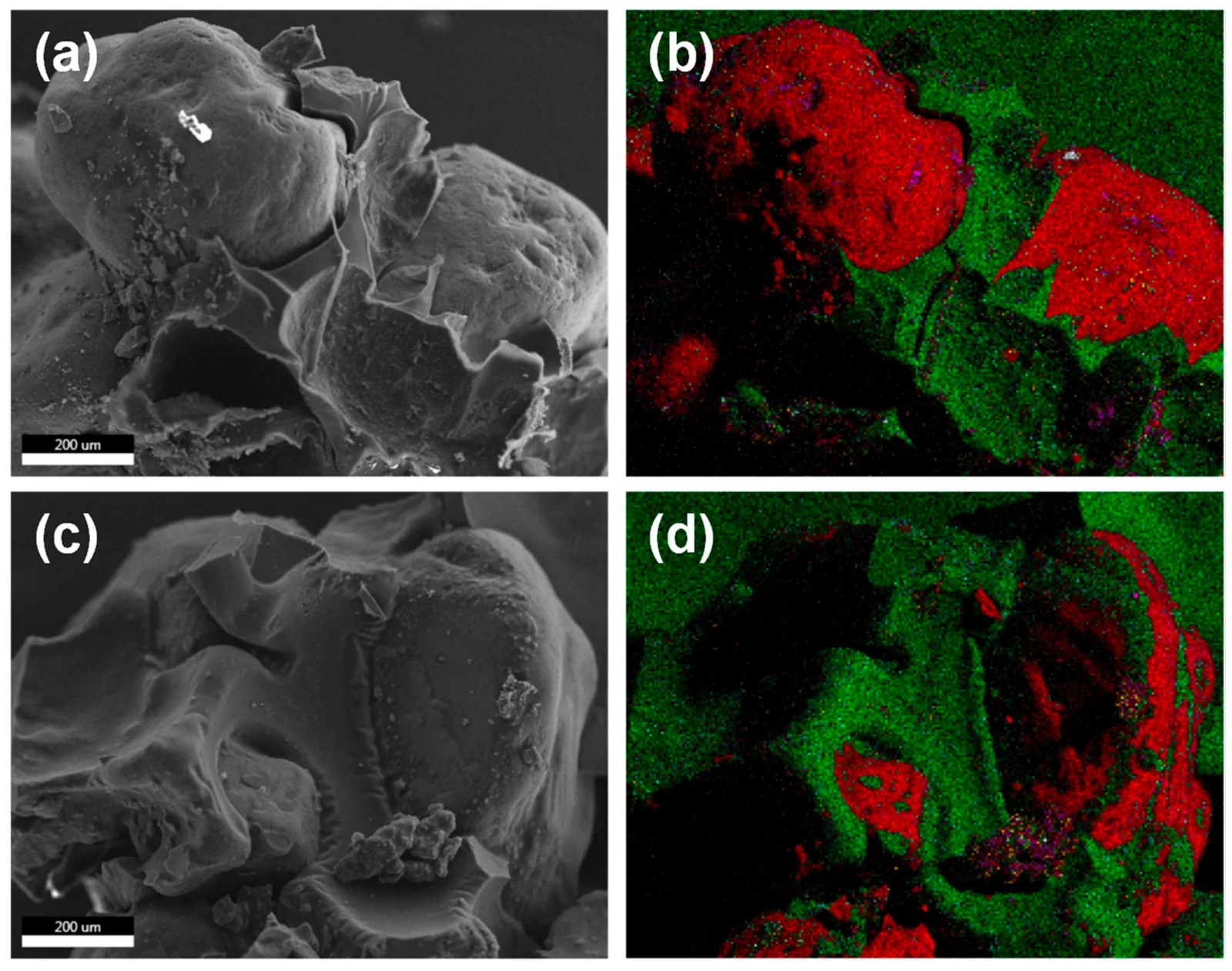
SEM micrographs (**a**,**c**) and corresponding EDS overlay maps (**b**,**d**) of the gel-consolidated sediment, showing the polymer gel network (Green, C) tightly encapsulating and bridging the inorganic sand grains (Red, Si).

The microstructural paradigm of the foam-templated gel consolidation was further investigated via SEM and EDS mapping (Figure 12). In stark contrast to bulk resin injection that indiscriminately fills interstitial voids, the foamed polymer gel selectively accumulates at inter-granular contact points to form robust pendular rings and bridging structures (Figure 12a,c). The elemental overlay and mapping profiles (Figure 12b,d) clearly demonstrate the spatial co-localization of the phases: the discrete inorganic quartz grains (Red, Si) are tightly encapsulated and bridged by a continuous, highly resilient film of the organic polymer gel network (Green, C). This interfacial-directed placement explains how the foam-templated gel system achieves a high compressive strength of ~6 MPa while simultaneously reducing chemical resin consumption by 55.6%. By confining the binder exclusively to grain junctions, the interstitial void space is highly preserved, maintaining a bulk porosity exceeding 45% and safeguarding the reservoir’s hydrodynamic permeability.

#### 2.4.2. Dynamic Evolution and Capillary Transport of Foam

**Figure 13:**
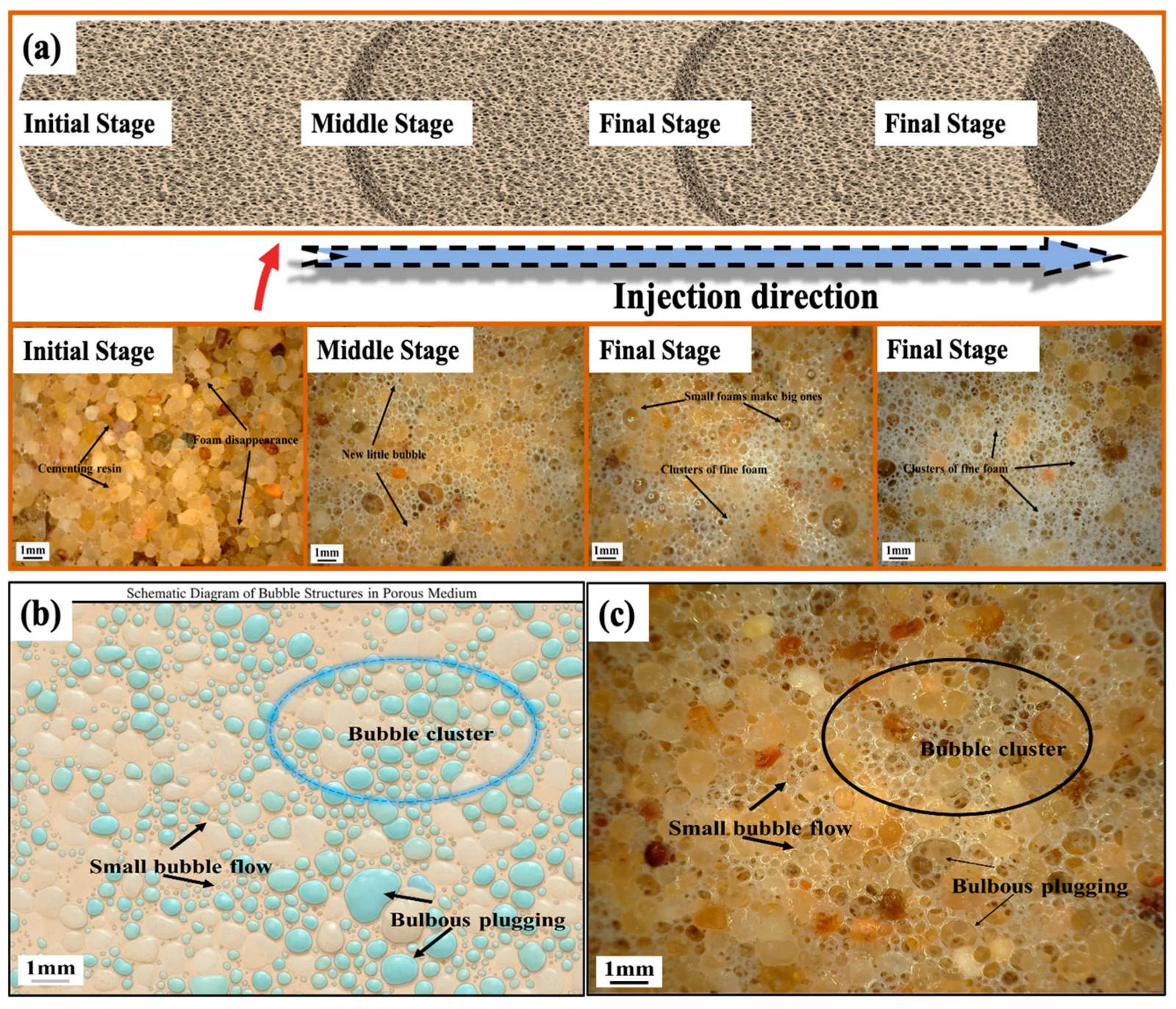
Dynamic evolution and capillary transport of the multiphase foam within the porous medium: (**a**) schematic illustrations and corresponding microscopic visual tracking of the multiphase flow stages; (**b**) mechanistic schematic and (**c**) corresponding microscopic visualization of localized colloidal behaviors, highlighting bubble accumulation, capillary-driven microbubble migration, and the Jamin effect-induced selective pore blocking.

To decode the transport behavior, the spatial evolution of the foam was monitored during injection. The foam undergoes four distinct stages governed by capillary thermodynamics (Figure 13):

(1) Interfacial Rupture & Wetting: At the injection front, strain-induced lamella rupture releases the resin, while surfactants alter surface wettability to strongly water-wet, facilitating precise capillary bridging at grain contacts.
(2) Lamella Division & The Jamin Effect: As bubbles enter narrow pore throats, the Jamin effect triggers structural hysteresis. Bubbles undergo “snap-off” and division, resulting in a bimodal dispersion (large bubbles >2 mm; microbubbles <1 mm).
(3) Shear-Induced Refinement: Deep within the matrix, intense shear forces promote continuous bubble necking, generating a dense population of fine emulsion-like foams.
(4) Sacrificial Templating: Upon cessation of flow, the foam occupies the medium as a stable colloidal network. Subsequent polymer gel cross-linking and gas evaporation leave behind a vast network of preserved capillaries, helping preserve an interconnected pore network and hydrodynamic conductivity.

Foam coarsening can destabilize bubbles during porous-media transport and consequently alter the mobility and spatial distribution of the transient resin-carrying template [38]. In the present formulation, BS-12, PVA, and nano-SiO_2_ help retard drainage and coarsening, thereby maintaining the foam template during resin redistribution. After placement and curing, mechanical stability is governed primarily by the cross-linked epoxy network rather than by the transient foam phase.

#### 2.4.3. Visual Sweep Efficiency and Viscous Fingering Suppression

**Figure 14:**
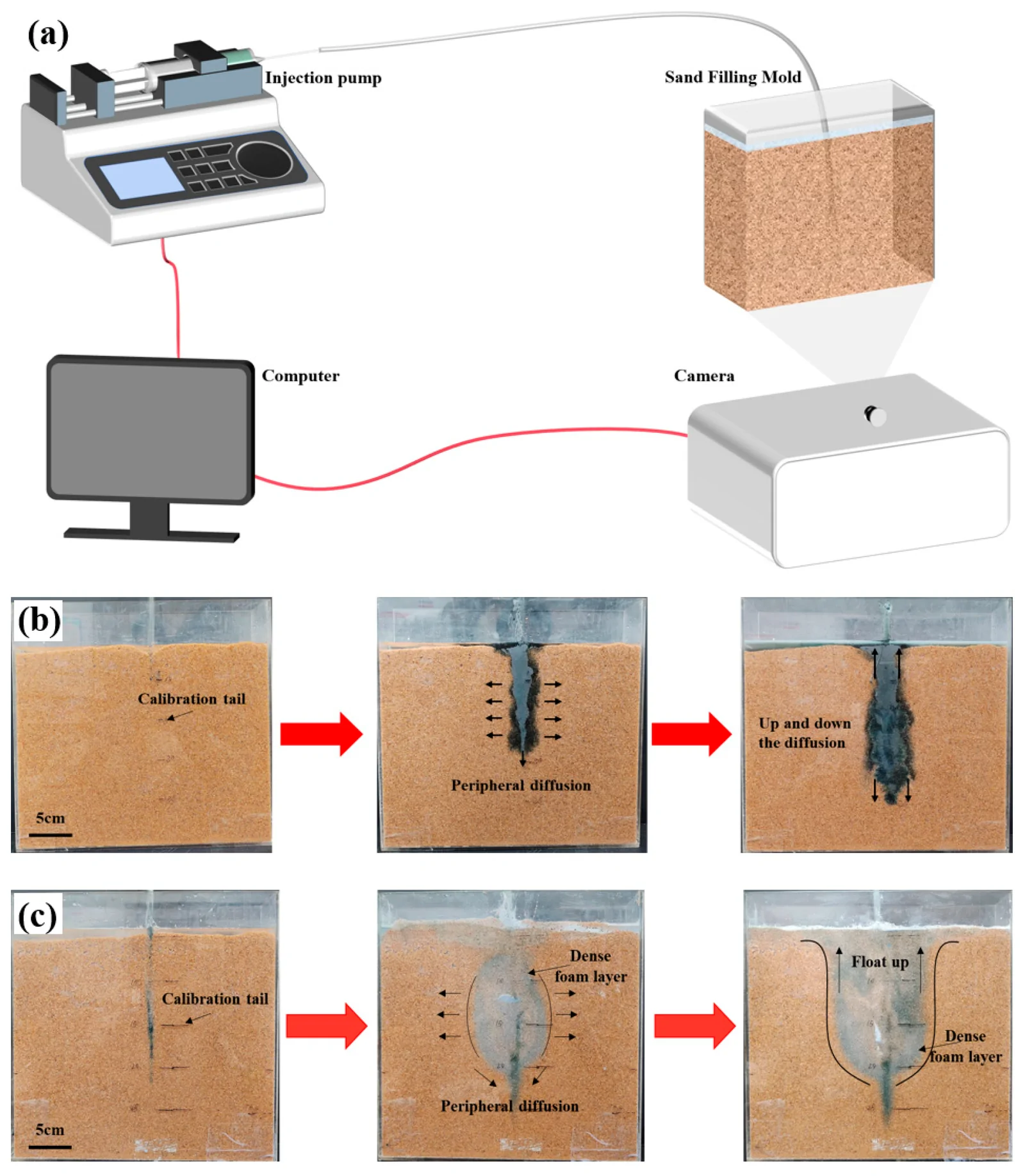
Macroscopic sweep efficiency and suppression of hydrodynamic instability: (**a**) transparent core-flooding visualization model; (**b**) bulk resin solution exhibiting severe gravity override and viscous fingering; (**c**) foam-templated polymer gel system demonstrating a uniform radial sweep and complete suppression of viscous fingering.

The macroscopic sweep efficiencies (Figure 14) exhibited stark contrasts:

Bulk Resin (Hydrodynamic Instability): Governed by Newtonian rheology, the bulk fluid exhibited severe gravity override and viscous fingering. It bypassed the majority of the matrix, channeling along the injection interface. Post-curing, it formed an irregular, localized mass (251.20 cm^3^), consolidating only 183.06 g of the matrix.

Foam Multiphase System (Interfacial Flow Control): In sharp contrast, the foam-templated gel system propagated as a uniform, spherical front. The enhanced apparent viscosity and reduced bulk density suppressed gravity override and eliminated viscous fingering. As the foam selectively accumulated in high-permeability zones, localized capillary bridging dynamically equalized the pressure gradient, ensuring a homogeneous radial sweep. This resulted in a uniform consolidated volume (530.66 cm^3^), securing 386.72 g of the matrix.

The foam-templated gel system consolidated 386.72 g of matrix compared with 183.06 g for the bulk resin system using the same polymer mass (50 g).

### 2.5. Pilot-Scale Engineering Validation

#### 2.5.1. Solid Entrainment Kinetics and Sand Control Efficacy

**Figure 15:**
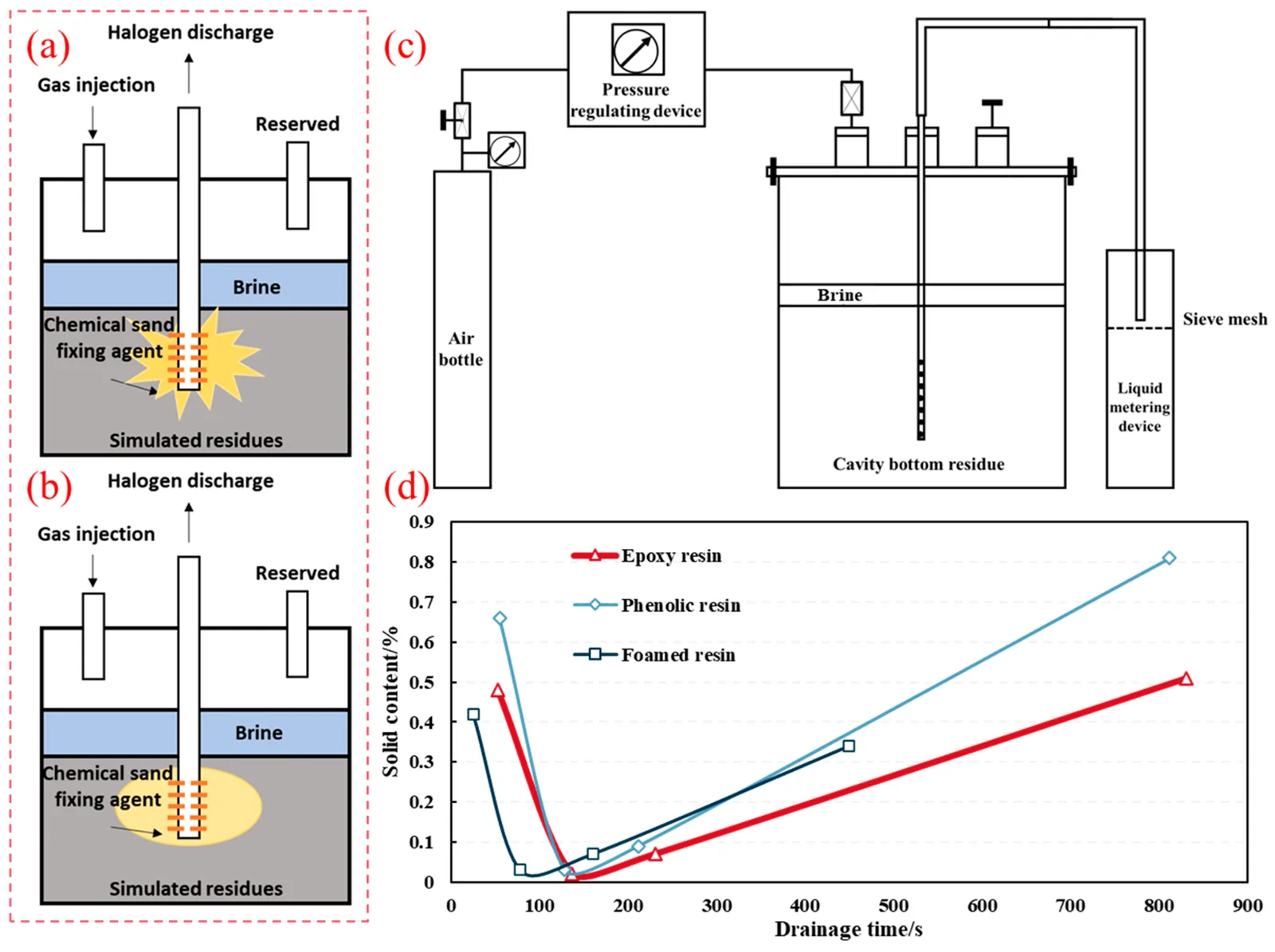
Macroscopic hydrodynamic simulation and solid entrainment kinetics: (**a**) multiphase displacement utilizing bulk resin; (**b**) hydrodynamic stabilization via the foam-templated system; (**c**) configuration of the 1:200 scaled physical flow simulation apparatus; (**d**) transient solid entrainment kinetics demonstrating enhanced granular retention by the multiphase foam.

The macroscopic fluid displacement experiments were systematically conducted across the three consolidated matrices (Table 2, Table 3 and Table 4; Figure 15d). Macroscopically, all three systems exhibited a consistent solid entrainment trend: an initial peak during primary breakthrough, followed by a sharp decline, and a marginal secondary increase as the mobile aqueous phase neared depletion. This trend reflects the shear-induced compaction of the granular skeleton under the applied gas pressure gradient.

Crucially, the multiphase foam-templated system achieved superior fluid discharge and solid retention. This performance is governed by two coupled mechanisms:

(1) Structural Porosity Preservation: The dynamic foam targets resin cross-linking exclusively at inter-granular contacts. This interfacial cementation preserves a highly interconnected void network (porosity > 45%), whereas traditional bulk resins suffer from localized pore clogging, drastically increasing flow resistance.
(2) Dynamic Flow Field Optimization (The Jamin Effect): During injection, the Jamin effect induces localized capillary resistance in large, high-permeability channels. This spontaneously redistributes the displacement fluid, ensuring a uniform radial sweep and eliminating the chaotic viscous fingering typical of bulk resin injection.

Validation against Industrial Standards: This macroscopic model rigorously bridges laboratory phenomena with field operations. Under simulated cavern conditions (0.1 MPa; 20 °C), the maximum solid entrainment rate for the foam-templated matrix was restrained below 0.5% (Table 4). This robust structural reliability, confirmed by extended validations using diverse granular matrices [14], supports the engineering transferability of the strategy.

#### 2.5.2. Microstructural Evidence of Interfacial Sludge Capture

**Figure 16:**
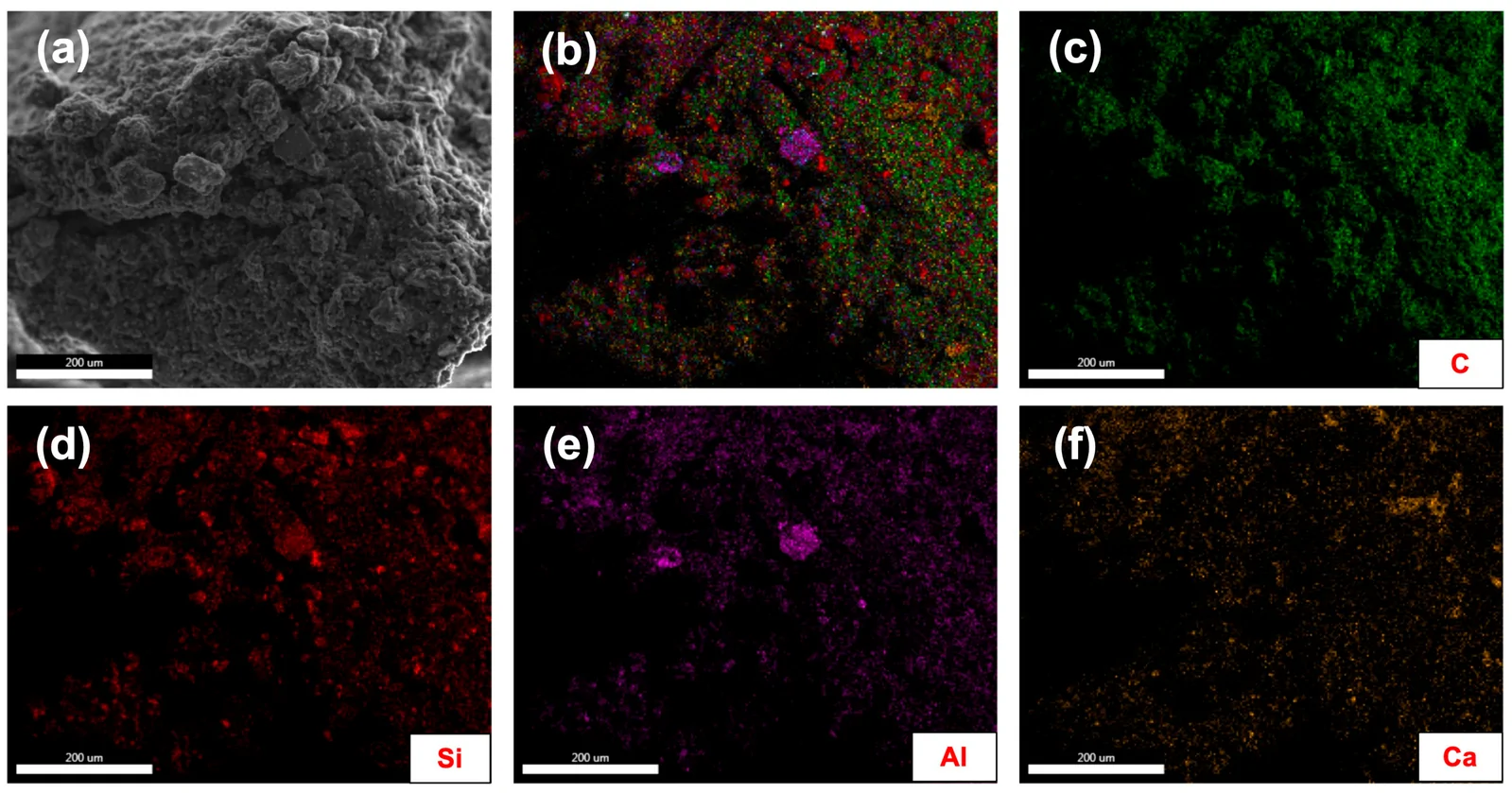
SEM micrograph (**a**) and corresponding EDS mappings (**b**–**f**) of the consolidated real salt cavern sludge: (**b**) all-element overlay; (**c**) C (polymer network); (**d**) Si (quartz/nano-SiO_2_); (**e**) Al (clay minerals); (**f**) Ca (anhydrite/dolomite fragments).

To validate the engineering applicability of this stabilization strategy in authentic field environments, the microstructural architecture of consolidated real salt cavern insoluble residues (sludge) was characterized (Figure 16). Unlike idealized quartz sand, real cavern sludge exhibits extreme mineralogical heterogeneity, poly-disperse particle sizes, and irregular morphology (Figure 16a). The EDS mapping profiles (Figure 16b–f) reveal that while silicon (Figure 16d, Si) represents the primary clastic skeleton, the matrix contains significant fractions of water-sensitive clay minerals (Figure 16e, Al) and anhydrite/dolomite fragments (Figure 16f, Ca) derived from salt cavern interbeds. Remarkably, the carbon mapping (Figure 16c, C) demonstrates that the polymer gel forms a continuous, resilient encapsulation membrane across these highly complex, multi-component geologic residues. This confirms polymer coverage across the heterogeneous residue matrix under the tested hypersaline conditions, successfully locking fine, easily mobilized particulates in place and restricting macroscopic sediment entrainment to strictly below 0.5% during high-rate fluid discharge.

**Figure 17:**
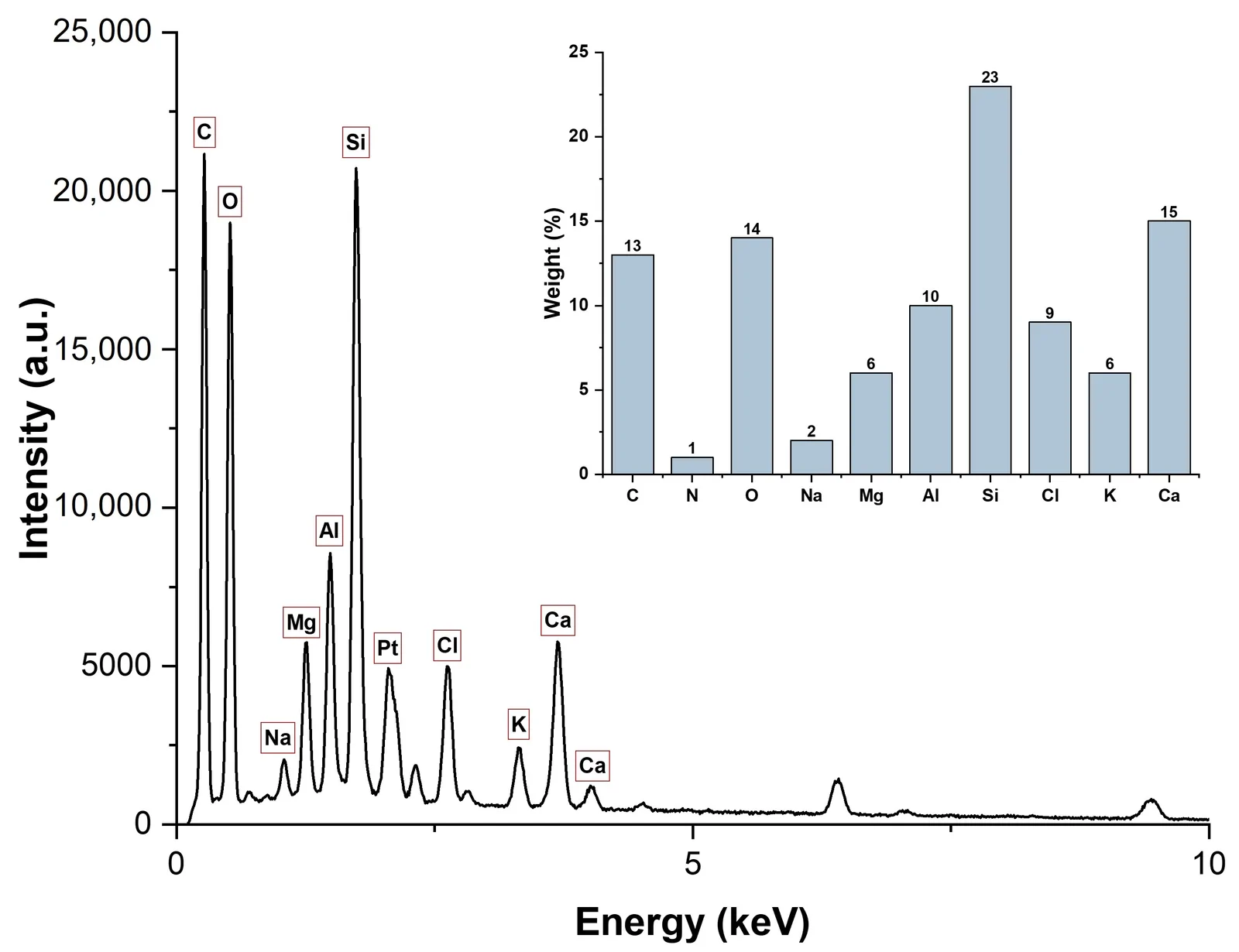
EDS spectrum and quantitative elemental composition of the consolidated real salt cavern sludge, confirming the interfacial capture of complex geological residues.

The quantitative elemental composition of the consolidated cavern sludge was further corroborated via EDS spectral profiling (Figure 17). The corresponding energy spectrum and numerical distribution confirm that inorganic geological elements dominate the bulk mass, led by silicon (23.0 wt%), calcium (15.0 wt%), and aluminum (10.0 wt%), which reflect the native quartz, carbonate/sulfate interbeds, and clay mineralogy of the target salt cavern formation. The detection of chlorine (9.0 wt%) and sodium (2.0 wt%) further reflects the persistent hypersaline brine background of the underground storage cavity. Despite the predominance of these complex inorganic and saline phases, the C signal accounted for approximately 13 wt% in the semi-quantitative EDS analysis. This quantitative distribution verifies that a relatively low mass fraction of interfacial-directed polymer is consistent with effective interfacial binding of the heterogeneous residues, providing a rigorous chemical basis for the exceptional geomechanical stability observed in large-scale physical simulations.

#### 2.5.3. Cyclic Hydrodynamic Stability

The radially distributed, cross-linked structural shell formed by the foam-templated fluid avoids creating adverse hydrodynamic bottlenecks. Governed by interfacial capillary limits, the consolidated shell maintains a controlled spatial thickness (2–3 cm) while CT-derived permeability estimate of approximately 1.18 D due to the absence of bulk pore-throat occlusion.

The macroscopic flow response demonstrated a negligible 3% cumulative increase in gas injection pressure (from 0.100 MPa to 0.103 MPa) across all cycles. The absence of injection interval blockage or localized pressure build-up supports cyclic hydrodynamic stability under the tested conditions and permeability retention, robustly securing the flow integrity of CCUS infrastructure under cyclic multiphase stress.

## 3. Conclusions

This study systematically developed and validated a foam-templated polymer gel consolidation strategy to mitigate fluid-induced sediment entrainment during the critical debrining phase of subsurface energy storage. By bridging microscopic multiphase fluid dynamics with macroscopic engineering simulations, the principal conclusions are derived:

(1) Engineered Resilience for Extreme Subsurface Conditions: A specialized nano-reinforced foam-templated polymer gel was formulated to withstand aggressive hypersaline (up to 24% NaCl) and elevated-temperature (20–80 °C) conditions of deep salt caverns. The zwitterionic surfactant (BS-12) effectively shields counterion interference, while the synergistic integration of silica nanoparticles and PVA constructs a highly resilient interfacial network. This engineering formulation ensures that the polymer gel template exhibits outstanding salt and thermal resistance, surviving the extreme geomechanical stresses of subterranean deployment.
(2) Paradigm Shift in Pore-Scale Flow Dynamics: The dynamic transport of the foam within the unconsolidated matrix fundamentally optimizes fluid placement. Governed by the Jamin effect and a dynamically modulated mobility ratio, the foamed system selectively blocks high-permeability channels and completely suppresses the viscous fingering inherent to conventional bulk resins. This foam-templated gel mechanism precisely directs cementation to grain-contact points, substantially reducing required chemical binder consumption by 55.6% while preserving a highly interconnected hydrodynamic network (porosity >45% and permeability of 1.18 D).
(3) Macroscopic Feasibility for Energy Storage Infrastructure: Using a 1:200 geometrically scaled, velocity-matched pilot model under controlled hydrodynamic conditions, the polymer gel strategy successfully restricted catastrophic sediment entrainment to strictly below 0.5%. Furthermore, the optimized multiphase sweep efficiency accelerated the macroscopic fluid discharge rate by 45.9%, confirming the system’s ability to safely and rapidly reclaim operational cavern space.

In summary, this research resolves a critical hydrodynamic bottleneck in salt cavern operations. By shifting the stabilization paradigm from “bulk fluid filling” to “foam-templated polymer gel templating,” this work provides a scalable, sustainable, and high-performance polymer gel solution. It offers a robust technological pathway for securing the structural integrity and maximizing the operational lifecycle of global CCUS and underground energy storage infrastructure.

## 4. Materials and Methods

### 4.1. Experimental Design and Hierarchical Validation Framework

To ensure scientific rigor, this study adopts a hierarchical experimental approach, transitioning from mechanistic elucidation to engineering validation:

1. Mechanistic Elucidation (Idealized Baseline): In the microscopic (Section 2.3) and visual flow (Section 2.4) experiments, we employ standardized quartz sand. This “Idealized Baseline” is essential to isolate fundamental interfacial mechanisms—such as colloidal stabilization and foam-templated gel sweep efficiency—from the inherent chemical noise of native clay and zeolite. By stripping away geochemical interference, we can clearly elucidate the intrinsic advantage of the polymer gel stabilization mechanism.
2. Engineering Validation (Representative Prototype): Subsequently, in the pilot-scale macroscopic simulation (Section 2.5), we utilize the Representative Engineering Matrix (the 4:4:2 multi-component synthetic residue). This model incorporates actual mineralogical heterogeneity and particle size distribution. By demonstrating that the interfacial gel mechanisms remain robust and effective under these complex, heterogeneous field conditions, we provide compelling evidence for the practical industrial applicability of our polymer gel consolidation strategy.

This dual-tier approach—combining mechanistic clarity with engineering realism—constitutes a rigorous, multi-scale verification framework that bridges theoretical interfacial science with large-scale geo-energy and CCUS infrastructure security.

### 4.2. Field Samples and Residue Preparation

Field-scale salt cores and insoluble geo-residues were harvested from typical continental salt deposits in China, focusing on the Jintan (Jiangsu Province) and Chuzhou (Anhui Province) regions. To ensure geological breadth, core samples were integrated across depth intervals from 920 to 2100 m. To prepare representative specimens for compositional analysis, a rigorous treatment protocol was conducted mimicking the field solution mining process. The native rock samples were first crushed and stirred evenly, followed by dissolution in distilled water to extract the primary argillaceous insoluble materials. Subsequently, the recovered sludges were placed in an oven and dried at 210 °C for 24 h to completely remove the water of crystallization, and then mechanically sieved (100–140 mesh) to obtain a standardized granular profile. The recovered materials were composited to obtain an engineering-representative residue rather than to resolve depth-dependent compositional variations. Initial petrophysical observations confirmed a completely unconsolidated, cohesionless granular architecture (Figure 2a). To align our pilot-scale hydrodynamic simulations with field-scale constraints, the Jintan Type III salt cavern was selected as the primary engineering prototype, as detailed in Table 5. These distinct regional provenances define the geomechanical boundary conditions for reservoir-equivalent formation reconstruction, directly mapping field-scale geological constraints onto the pilot-scale apparatus design.

The quantitative geomechanical dimensions of these target reservoirs, detailed in Table 5, explicitly reveal the massive scale of in situ residue accumulation (up to 120.9 m in thickness). These severe boundary conditions directly necessitate and guide the geometric scaling of our macroscopic physical simulation.

### 4.3. Mineralogical and Particle-Size Characterization

The mineralogical composition of the 150-mesh powdered particulate was semi-quantitatively evaluated by X-ray diffraction (XRD, Empyrean, Malvern Panalytical, Almelo, The Netherlands) using MDI Jade (version 6.5, Materials Data, Inc., Livermore, CA, USA) software and the ICDD PDF-2 database. Phase proportions were estimated using the Reference Intensity Ratio (RIR) method.

To transcend the limitations of conventional idealized models, we formulated a mineralogically-matched synthetic residue. This multi-component approach ensures: (1) Geochemical Fidelity, where the incorporation of zeolite and clay fractions replicates the surface chemistry and reactive sites of in situ residues, subjecting polymer gel cementation to realistic mineral-fluid interaction conditions; and (2) Morphological Realism, where the inclusion of fines accounts for the heterogeneous frictional properties, cohesive tendencies, and particle migration behaviors inherent in actual salt cavern sediments.

### 4.4. Materials and Candidate Formulations

All chemical agents utilized in this study were purchased from Aladdin Biochemical Technology Co., Ltd. (Shanghai, China) and carefully selected to engineer a highly resilient multiphase system capable of withstanding the aggressive hypersaline and elevated-temperature conditions characteristic of deep salt cavern energy storage.

(1) Continuous Polymer Matrix (Structural Binder): Thermosetting phenolic, furan, urea-formaldehyde, and bisphenol-A type epoxy resins (E51) were evaluated as the continuous load-bearing phase. To initiate robust macromolecular cross-linking and ensure long-term geomechanical stability within the storage formation, specific curing agents including a silane coupling agent (KH550), polyetheramine (D230), and phenolicamines (NX-2003, NX-2003D) were employed.
(2) Amphiphilic Surfactants (Mobility Control Agents): To generate a stable gas–liquid interface capable of resisting severe double-layer compression in saturated brine environments, a series of surfactants was screened. This included alkyl phenol polyoxyethylene ether-10 (OP-10, nonionic), polyoxyethylene sorbitol monooleate (Tween 80, nonionic), sodium dodecyl sulfate (SDS, anionic), dodecyl dimethylamine ethyl lactone (BS-12, zwitterionic betaine), and lauramidopropyl hydroxysulfobetaine (LHSB, zwitterionic).
(3) Nanoparticle and Polymeric Stabilizers (Interfacial Reinforcement): To dramatically enhance the viscoelasticity of the liquid lamellae and prevent phase separation during the dynamic injection process, hydrophilic silica nanoparticles (nano-SiO_2_, 30 nm, ≥99.8% purity) were introduced to form rigid structural barriers at the interface. Furthermore, various water-soluble polymers, including polyethylene glycol (PEG), hydroxyethyl cellulose (HEC), and polyvinyl alcohol (PVA 1788 and 1750), were investigated for their capacity to construct cohesive hydrogen-bonded networks, ensuring the foam template maintains structural integrity under the high-pressure dynamics of cavern debrining.

The formulation of a multiphase consolidation fluid for subsurface applications requires a polymer matrix that exhibits not only favorable rheological properties for interfacial stabilization but also robust structural integrity upon cross-linking. Traditional thermosetting systems, including phenolic, furan, and epoxy resins, exhibit extreme hydrophobicity. To effectively disperse these precursors within an aqueous foam network, intermediate oil-in-water (O/W) emulsions must first be synthesized.

According to previous interfacial investigations [43], OP-10 was selected as the optimal nonionic emulsifier. To establish a baseline colloidal performance, O/W emulsions were formulated comprising 10 g of the respective resin, 0.6 g of OP-10, 0.5 g of SDS (as the primary foaming agent), and 8 mL of the aqueous phase.

### 4.5. Foam Generation and Stability Evaluation

The multiphase dispersion capability and transient stability of the complex fluid were evaluated using a high-shear mechanical dispersion method. A predefined volume of the polymer-surfactant colloidal solution was subjected to high-speed shearing (6000 rpm) for 1 min to dynamically generate the foam template. The resulting multiphase mixture was promptly transferred to a graduated cylinder to monitor the macroscopic drainage kinetics.

The initial foaming volume (V_0_) quantifies the system’s absolute foamability (interfacial generation capacity), while the half-life (t_1/2_) signifies the resistance of the liquid film against gravity-driven drainage and capillary suction (interfacial stability). To holistically evaluate the colloidal performance, a comprehensive colloidal stability index (F_c_) was calculated as the product of these two dynamic parameters [44]:

(1) F_c_ = V_0_·t_1/2_

where

F_c_—Foam comprehensive value, mL·min;
V_0_—Foaming volume, mL;
t_1/2_—Half-life of the solution, min.

While fundamental physicochemical parameters—such as surface tension and interfacial rheology—govern micro-scale coalescence kinetics, $F_{c}$ is adopted here as a practical, macroscopic engineering metric. Accordingly, $F_{c}$ is used here for comparative engineering screening rather than as a complete physicochemical descriptor of foam stability. It effectively integrates absolute generation capacity and temporal longevity, providing a practical basis for comparing formulations intended for macroscopic injectivity and sweep control in large-scale porous media.

### 4.6. FTIR and SEM-EDS Characterization

To fundamentally understand how the optimized foam-templated system achieves exceptional geomechanical stability and environmental robustness prior to sediment contact, the chemical cross-linking structure and microscopic morphology of the pure cured gel matrix were systematically investigated via FTIR spectroscopy (VERTEX 80v, Bruker, Ettlingen, Germany) and SEM-EDS mapping (GeminiSEM 300, Carl Zeiss, Oberkochen, Germany).

To further evaluate the structural uniformity and environmental robustness of this cross-linked network under hypersaline subterranean conditions, SEM coupled with EDS elemental mapping was conducted on the cured matrix.

### 4.7. Core Consolidation and Compression Testing

Uniaxial compression tests were conducted on cylindrical core specimens with a diameter of 25 mm and a height of 50 mm. The specimens were loaded along the axial direction under displacement-controlled quasi-static conditions at a loading rate of 0.5 mm·min^−1^. Axial strain was calculated as the platen displacement divided by the initial specimen height. Failure was defined as the peak compressive stress followed by a pronounced stress decrease and/or visible macroscopic cracking. One specimen was tested for each sample type; therefore, the reported strength represents a characteristic value rather than a statistical average, and variability statistics were not calculated. The representative stress–strain curve and post-failure core morphology are presented in Figure 8.

To elucidate the interfacial templating mechanisms at the pore scale, a custom-designed multiphase core-flooding apparatus was utilized (Figure 8a,b). Consistent with our hierarchical research strategy, standardized quartz sand (0.15–0.45 mm) was employed as the “Idealized Baseline” matrix. This choice deliberately isolates core-scale interfacial mechanics—specifically, the preferential polymer gel deposition at grain contact points—from the chemical noise and optical opacity inherent in field-retrieved heterogeneous sediments.

The experimental protocol was designed to precisely simulate the in situ interfacial physical chemistry and multiphase displacement processes:

(1) Granular Matrix Preparation: 20 g of standardized quartz sand (0.15–0.45 mm) was packed into a transparent observation column under controlled mechanical compaction to establish a reproducible, unconsolidated porous medium with uniform bulk density.
(2) Interfacial Modification: The matrix was initially saturated with 10 mL of hypersaline brine to establish the baseline fluid saturation. Subsequently, 10 mL of a prepad fluid (10% coupling agent solution) was injected to chemically modify the particulate wettability and enhance subsequent interfacial adhesion.
(3) Multiphase Injection: The optimized foam-templated polymer gel (mass ratio of epoxy resin: emulsifier: foaming agent: polymer stabilizer: nanoparticle: water = 100:6:4:4:1:80) was dynamically injected. As a control, a bulk single-phase epoxy resin solution (resin to curing agent ratio of 3:1) was also evaluated.
(4) Isothermal Fixation: The saturated core was sealed and subjected to isothermal curing at 60 °C for 48 h to complete the macromolecular polymerization and morphologically ’freeze’ the granular skeleton.

### 4.8. Micro-CT Acquisition and Analysis

The fundamental flaw of traditional bulk resin injection lies in its catastrophic impact on the reservoir’s hydrodynamic conductivity. To elucidate the distinct morphological consolidation paradigms at the pore scale, the cross-linked cores were subjected to petrophysical evaluation and high-resolution X-ray micro-computed tomography (Micro-CT). Micro-CT scanning was performed at 120 kV and 140 mA, with a reconstructed slice thickness of 0.625 mm. A central region of interest (ROI) of each specimen was selected for tomographic reconstruction, pore segmentation, and pore-network analysis. The resulting porosity and permeability values were derived from the reconstructed CT models and should therefore be regarded as CT-based estimates. Formal REV analysis, replicate CT scans, uncertainty quantification, and independent permeability validation were not performed.

### 4.9. Visual Multiphase-Flow Experiments

To translate these colloidal interactions into macroscopic flow behavior, a visual core-flooding model was utilized. Consistent with our hierarchical validation framework (Section 2.4), this visual model employs standardized quartz sand as an “Optical Baseline” to isolate fundamental transport mechanics from the light-scattering interference of native field residues.

### 4.10. Pilot-Scale Apparatus, Sediment-Entrainment, and Cyclic Stability Tests

To bridge interfacial mechanisms with field-scale applications, a pilot-scale hydrodynamic model was engineered to simulate gas-driven brine discharge. Utilizing the Jintan Type III salt cavern as the prototype (Table 5; 180-m height, 59-m residue thickness, 86-m equivalent diameter), a 1:200 geometric scaling was adopted. The custom cylindrical pressure vessel (60-cm inner diameter, 90-cm height) precisely scales the vertical profile, accommodating a 29.5-cm sediment bed and the structurally necessary fluid column. To mitigate artificial rigid-wall boundary effects and reduce wall constraints on radial flow, the vessel’s 60-cm diameter deliberately exceeds the 43-cm scaled horizontal flow field, providing an annular buffer of approximately 8.5 cm on each side. The porous medium was reconstructed using ~200 kg of the 4:4:2 particle-size blend to rigorously replicate subterranean heterogeneity.

A central extraction conduit (8-mm OD, 6-mm ID) was pre-installed into the matrix to simulate the production wellbore, eliminating mechanical penetration artifacts. To reproduce a field-relevant hydrodynamic boundary, the outlet velocity range was selected according to typical field operations (216-mm open-hole, 100.54-mm conduit ID). Standard field injection rates (30–120 m^3^/h) generate exit velocities of 1.0–4.2 m/s; correspondingly, the pilot-scale apparatus was engineered to deliver continuous velocities of 0–5.0 m/s. This velocity matching provides a controlled hydrodynamic condition for comparing the consolidation systems. However, because the Reynolds, Bond, and Froude numbers and the pressure gradient were not simultaneously matched, the model does not represent complete dynamic similarity with the field-scale cavern.

The experimental apparatus (Figure 15c) integrates gas regulation, pressure monitoring, and volumetric collection. The core displacement protocol proceeded as follows:

(1) Matrix Saturation: The simulated cavern cell was packed with the multi-component synthetic blend and saturated with 24% NaCl brine, establishing fluid-solid equilibrium.
(2) Hydrodynamic Templating: A 5-pore-volume slug of the respective consolidation fluid was dynamically injected. Driven primarily by hydrodynamic pressure gradients, the fluid selectively permeated the unconsolidated matrix.
(3) Isothermal Fixation: The sealed system underwent isothermal cross-linking at 20 °C for 72 h under simulated subterranean conditions.
(4) Multiphase Displacement: High-pressure nitrogen (0.1 MPa) was continuously injected to hydrodynamically drive the aqueous phase out through the extraction conduit.
(5) Effluent Monitoring: Transient discharge time, cumulative fluid volume, and entrained solid mass were quantitatively recorded until flow cessation.

To validate the long-term structural fatigue resistance against operational stresses, a sequential 10-cycle multiphase stress test was conducted.

## Figures and Tables

**Table 1:** Interfacial generation and drainage kinetics of various multiphase polymer systems.

Resin Type	Phenolic Resin	Furan Resin	Epoxy Resin	Urea-Formaldehyde Resin
Foaming volume V_0_/mL	98	127	69	110
Half-life period t_1/2_/min	20.52	14.22	99.5	58.43
Composite foam value F_c_/mL·min	2010.96	1805.94	6865.5	6427.30

**Table 2:** Hydrodynamic displacement data for the single-phase epoxy-consolidated matrix.

Number	Drain Time/s	Drain Time Accumulated/s	Drain Volume/mL	Mass of Solid/g	Solid Content/%
1	52.78	52.78	2000	9.64	0.48
2	84.2	136.98	2000	0.40	0.02
3	93.94	230.92	2000	1.41	0.07
4	600	830.92	600	3.06	0.51

**Table 3:** Hydrodynamic displacement data for the single-phase phenolic-consolidated matrix.

Number	Drain Time/s	Drain Time Accumulated/s	Drain Volume/mL	Mass of Solid/g	Solid Content/%
1	55.35	55.35	2000	13.14	0.66
2	73.29	128.64	2000	0.61	0.03
3	83.72	212.36	2000	1.80	0.09
4	600	812.36	1150	9.32	0.81

**Table 4:** Hydrodynamic displacement data for the multiphase foam-consolidated matrix.

Number	Drain Time/s	Drain Time Accumulated/s	Drain Volume/mL	Mass of Solid/g	Solid Content/%
1	29.92	29.92	2000	8.41	0.42
2	39.62	69.54	2000	0.62	0.03
3	81.63	121.25	2000	1.15	0.05
4	357.48	439.11	1500	5.12	0.34

**Table 5:** Geological and structural boundary parameters of typical target salt cavern gas storage facilities in China, serving as engineering prototypes for macroscopic hydrodynamic scaling.

Target Field (Region)	Cavern Type Well Name	Cavern Bottom Depth (m)	Total Cavern Height (m)	Basal Residue Height (m)	Calculated Radius (m)
Jintan (Jiangsu)	Type I	1150	110	40	38
	Type II	920	130	54	18
	Type III	1030	180	59	43
Chuzhou (Anhui)	Zhangxing	1600	200	120.7	39
	Yanghuai	2100	200	120.9	35

## Data Availability

The data presented in this study are available on request from the corresponding author.
